# ﻿*Fungi*: Pioneers of chemical creativity – Techniques and strategies to uncover fungal chemistry

**DOI:** 10.3897/imafungus.16.142462

**Published:** 2025-03-07

**Authors:** Hedda Schrey, Christopher Lambert, Marc Stadler

**Affiliations:** 1 Department Microbial Drugs (MWIS), Helmholtz-Centre for Infection Research, 38124 Braunschweig, Germany Helmholtz-Centre for Infection Research Braunschweig Germany; 2 Institute of Microbiology, Technische Universität Braunschweig, Spielmannstraße 7, 38106 Braunschweig, Germany Technische Universität Braunschweig Braunschweig Germany

**Keywords:** Analytics, antibiotics, bioprospecting, biosynthesis, chemotaxonomy

## Abstract

Natural product discovery from fungi for drug development and description of novel chemistry has been a tremendous success. This success is expected to accelerate even further, owing to the advent of sophisticated technical advances of technical advances that recently led to the discovery of an unparalleled biodiversity in the fungal kingdom. This review aims to give an overview on i) important secondary metabolite-derived drugs or drug leads, ii) discuss the analytical and strategic framework of how natural product discovery and drug lead identification transformed from earlier days to the present, iii) how knowledge of fungal biology and biodiversity facilitates the discovery of new compounds, and iv) point out endeavors in understanding fungal secondary metabolite chemistry in order to systematically explore fungal genomes by utilizing synthetic biology. An outlook is given, underlining the necessity for a collaborative and cooperative scenario to harness the full potential of the fungal secondary metabolome.

## ﻿Introduction

This review is dedicated to Dr. David J. (“Dave”) Newman, formerly Chief of the Natural Products Branch (NPB) in the Developmental Therapeutics Program at the National Cancer Institute (NCI) in Frederick, Maryland. The current review was first thought to be a book chapter but since the book was not going to materialize after some time, we have decided to dedicate it to Dave as a paper. He has (fide Scopus, Nov. 2024) published over 190 papers, which received more than 36.000 citations and his h index is 58. His most well-known contributions are the reviews on the importance of natural products among the therapeutic agents that got published in the Journal of Natural Products (cf. Newman and Cragg 2020 for the latest update of this series). Dave is still active in the community even after his official retirement in 2015. We deeply respect his contribution to the field of natural product research and wish him all the best.

We will here outline the importance of fungi as sophisticated creators of secondary metabolites. In the course of their evolution, fungi have become highly creative and elaborate producers of such natural products, which display a high degree of structural diversity and novelty. This is commonly attributed to their immobile lifestyles and constant competition against other organisms (Spiteller 2008; Bills and Gloer 2016). Their unique and complex metabolites and biological effects have fascinated generations of natural product researchers of different fields in the past and present. For over a century, fungi served as a treasure trove for the exploration of natural products for the benefit of humankind. As early as 1893, mycophenolic acid (**1**), produced by the mold *Penicilliumbrevicompactum*, was discovered as the first antibiotic (Gosio 1893; Gosio 1896). Although **1** was not successfully commercialized as an antibacterial drug due to its toxic side effects, the semisynthetic derivative mycophenolate mofetil (**2**) has been launched to the market as a potent immunosuppressant about one hundred years later (Bentley 2000, Table 1). After WW II, the discovery of the penicillins (**3**) and their subsequent production in industrial scale opened the door to the “golden era of antibiotics” persisting for several decades (Hutchings et al. 2019). To date, thousands of secondary metabolites have been discovered with a wide range of biological properties. Some of them are usable for human benefit, while ingestion of others, such as mycotoxins that contaminate food, may have potentially fatal consequences (Ráduly et al. 2020). Between the late 1930’s and the late 1950’s, noteworthy anti-infective agents such as cephalosporin C (**4**) (Newton and Abraham 1955), pleuromutilin (**5**) (Kavanagh et al. 1951), fusidic acid (**6**) (Godtfredsen et al. 1962), and griseofulvin (**9**) (Oxford et al. 1939) were discovered from fungi. Further examples of early described compounds are the illudins (**10**–**11**) (Anchel et al. 1950), which have been studied extensively for their cytotoxicity. Over the next three decades, other important fungal metabolite classes were discovered, such as the cytochalasins (**12**–**14**) (Aldridge et al. 1967), myriocin (**15**) (Kluepfel et al. 1972 and Mapook et al. 2022), cyclosporin A (**16**) (Rüegger et al. 1976), the statins (**17**–**19**) (Endo 2008), the echinocandins (**20**–**25**) (Benz et al. 1974; Schwartz et al. 1989; Iwamoto et al. 1994; Hüttel 2021) as well as the strobilurins (**26**–**27**) (Anke et al. 1977; cf. Table 1).

**Table 1.** Important natural products from fungi and common applications (reviewed by Bills and Gloer 2016; Hutchings et al. 2019; Newman and Cragg 2020).

class	discovery reported	producing organism	introduced into use	example	use
mycophenolic acids	1893, **myco-phenolic acid** (**1**)^a^	*Penicilliumbrevicompactum*	1995	**mycophenolat-mophetil** (**2**, semi-synthetic derivative of mycophenolic acid)	immuno-suppressive (prevention of organ rejection following kidney, liver, heart transplant)
kojic acid	1907, **kojic acid**^b^	*Aspergillus* (*flavus* var.) *oryzae*	1955	**kojic acid**	antioxidant in cosmetic products used for skin lightening in Asian countries
ergot alkaloids	1918, **ergotamine**^c^	*Clavicepspurpurea*, *C.fusidormis*, *C.paspali*	1921	e.g. **ergotamine tartrate**, **dihydroergotamine mesylate**	vasoconstrictor (third-line therapy of migraine)
	1935, **ergometrine** (**27**)^d^	*Clavicepspurpurea*, *C.fusidormis*, *C.paspali*	1936	**methylergometrin** (semisynthetic derivative of ergometrine)	uterotonic (treatment of postpartum haemorrhage)
	1967, **ergocryptine**^e^	*Clavicepspurpurea*, *C.fusidormis*, *C.paspali*	1975	**bromocriptine** (semi-synthetic derivative of ergocyptine)	treatment of reproductive disorders, Parkinson’s disease
*β*-lactams	1929, **penicillin G** (**3**)^f^	*Penicilliumrubens*	1943	penicillins e.g. **amoxicillin** (**29**, semi-synthetic derivative of penicillin G)	antibiotic (against Gram-positive and Gram-negative bacteria)
	1945, **cephalo-sporin C** (**4**)^g^	*Acremoniumchrysogenum*	1964	cephalosporins e.g. **cephalotin** (**33**, semi-synthetic derivative of cephalosporin C)	antibiotic (against Gram-positive and Gram-negative bacteria)
phallotoxins	1937, **phalloidin**^h^	*Amanitaphalloides*	-	**phalloidin** ^ae^	F-actin staining, fluorescence microscopy
gibberellins	1938, **gibberellic acid**^i^	*Fusariummoniliforme*	late 1950’s	**gibberellic acid**	phytohormone for plant development used as biochemical in agriculture
griseofulvin	1939, **griseofulvin**^j^	*Penicilliumgriseofulvum*	1959	**griseofulvin**	antimycotic (therapy of skin, hair, and nails)
illudins	1950, **illudins M** (**10**) and **S** (**11**)^k^	*Omphalotusilludens*	under development	**irofulven**^af^ (semi-synthetic analogue of illudin S)	anticancer (failed in clinical trials)
pleuro-mutilins	1951, **pleuro-mutilin** (**5**)^l^	*Clitopiluspasseckerianus*	2019	e.g. **lefamulin** (**37**, semisynthetic derivative of pleuromutilin)	antibiotic
wortmannin	1957, **wortmannin**^m^	*Talaromyceswortmannii*	-	**wortmannin** ^ag^	anticancer, Pl3K-inhibitor in cell assays (failed in clinical trials)
brefeldins	1958, **brefeldin A**^n^ (decumbin)	*Penicilliumdecumbens*	-	**brefeldin A** ^ah^	biochemical tool for the study of membrane trafficking and secretion
psilocybin	1959, **psilocybin** (**38**) ^o^	*Psilocybe* spp.	under development	**psilocybin** (**38**) ^ai^	major depressive disorder (not yet generally approved)
fusidic acid	1962, **fusidic acid** (**6**)^p^	*Ramulariacoccinea*	1962	**fusidic acid** (**6**)	antibiotic (against Gram-positive bacteria including methilicin-resistant *Staphylococcusaureus*)
zearalenones	1962, **zearalenone**^q^	*Fusariumgraminearum*	1969	***α*-zearalenol** (semi-synthetic derivative of zearaleone)	anabolic agent used as growth promoter in beef cattle and sheep in North America
cytochalasins	1967, **cytochalasin A** (**12**) and **B** (**13**)^r^	*Pyrenophoradematioidea*	development aborted	e.g. **cytochalasin B** (**13**)^aj^	antiviral, biochemical tool for the study of cell division and cell motility
mizoribine	1971, **mizoribine**^s^ (bredinin)	*Penicilliumbrefeldianum*	1984	**mizoribine**	immuno-suppressive in Japan, Korea, and China (used for renal transplants)
myriocins	1972, **myriocin** (**15**)^t^	*Melanocarpusalbomyces*, *Isariasinclairii*	2010	**fingolimod** (**35**, semi-synthetic derivative of myriocin)	immuno-suppressive (treatment of multiple sclerosis)
cyclosporin	1976, **cyclosporin A** (**16**)^u^	*Tolypocladiuminflatum*	1983	**cyclosporin A** (**16**)	immuno-suppressive (prevention of organ transplant and tissue graft rejection)
statins	1976, **mevastatin** (**18**, ML-236B) ^v^	*Penicilliumcitrinum*	1991	**pravastatin** (**19**, semisynthetic derivative of mevastatin)	cholesterol lowering
	1978, **lovastatin** (**17**, mevinolin) ^w^	*Monascusruber*; *Aspergiluusterreus*	1987	e.g. **lovastatin** (**17**), **simvastatin** (semisynthetic derivative of lovastatin)	cholesterol lowering
echino-candins	1974, **echinocandin B** (**20**)^x^	*Aspergillusdelacroxii*	2006 / 2023	**anidulafungin** (semi-synthetic derivative of echinocandin B) / **rezafungin** (**21**, analog of anidulafungin)	antimycotic (first-line therapy against systemic infections)
	1989, **pneumo-candin A_0_**^y^ (L-671,329)	*Glarealozoyensis*	2001	**caspofungin** (semi-synthetic derivative of pneumocandin B_0_)	antimycotic (first-line therapy against systemic infections)
	1994, **FR901379**^z^ (**24**, WF11899A)	*Coleophomaempetri*	2005	**micafungin** (semi-synthetic derivative of FR901379)	antimycotic (first-line therapy against systemic infections)
strobilurins	1977, **strobilurin A** (**26**)^ab^	*Strobilurustenacellus*	1996	e.g. **azoxystrobin** (synthetic derivative)	agro-fungicide
cyclodepsi-peptides	1992, **PF1022 A**^ac^	*Rosellinia* spp.	2005	**emodepsid** (semi-synthetic derivative of PF1022 A)	anthelmintic, veterinary medicine
enfuma-fungins	2000, **enfuma-fungin** (**40**)^ad^	*Hormonemacarpetanum*	2020	**ibrexafungerp** (**39**, semisynthetic derivative of enfanufungin)	antimycotic (systemic infections)

^a^ (Gosio 1893; Gosio 1896); ^b^ (Saito 1907); ^c^ (Stoll 1918); ^d^ (Stoll 1935); ^e^ (Amici 1969); ^f^ (Fleming 1929); ^g^ (Newton and Abraham 1955); ^h^ (Lynen and Wieland 1938); ^i^ (Yubata and Sumiki 1938); ^j^ (Oxford et al. 1939); ^k^ (Anchel et al. 1950); ^l^ (Kavanagh et al. 1951); ^m^ (Brian et al. 1957); ^n^ (Singleton et al. 1958); ^o^ (Hofmann et al. 1958); ^p^ (Godtfredsen et al. 1962); ^q^ (Stob et al. 1962); ^r^ (Aldridge et al. 1967); ^s^ (Mizuno et al. 1974); ^t^ (Kluepfel et al. 1972); ^u^ (Rüegger et al. 1976); ^v^ (Endo et al. 1976); ^w^ (Alberts et al. 1980); ^x^ (Benz et al. 1974); ^y^ (Schwartz et al. 1989); ^z^ (Iwamoto et al. 1994); ^ab^ (Anke et al. 1977); ^ac^ (Sasaki et al. 1992); ^ad^ (Peláez et al. 2000); ^ae^ (Wulf et al. 1979); ^af^ (Gheysen et al. 2020); ^ag^ (Liu et al. 2005); ^ah^ (Chardin and McCormick 1999); ^ai^ (Kargbo 2020); ^aj^ (Cooper 1987).

**Figure 1.**
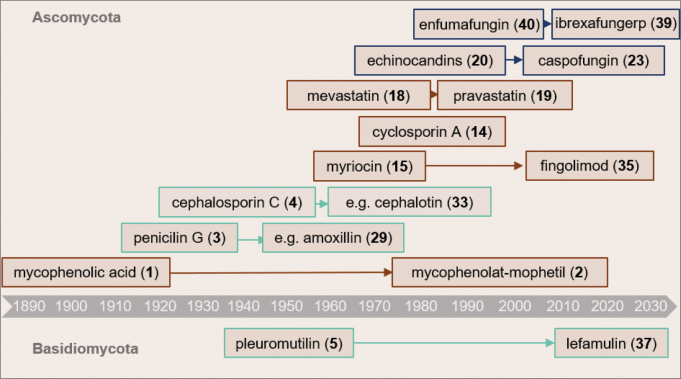
Timeline of compounds developed into now Blockbuster drugs from *Ascomycota* (above) and *Basidiomycota* (below) together with their semisynthetic derivatives. The compounds are used as antibacterials (green line), antifungal compounds (blue line), and other indications (orange line).

These discoveries have provided valuable lead structures and pharmacophores for medicinal chemistry, contributing to the development of numerous drugs and market blockbusters (Fig. 1). According to the World Health Organization’s List of Essential Medicines in 2019, several fungal-derived metabolites are deemed essential for human healthcare. For instance, ergometrine (**27**), first isolated by Stoll in 1935, is utilized as an uterotonic following childbirth (Stoll 1935; McDonald et al. 2004). Additionally, griseofulvin (**9**) serves as an antimycotic agent for the treatment of dermatophytoses (Petersen et al. 2014) and the semisynthetic *β*-lactam antibiotics like ampicillin (**28**), amoxicillin (**29**), cefazolin (**30**), cefalexin (**31**), and ceftazidime (**32**), remain crucial antibacterial blockbusters, with a current annual market share exceeding 20 billion USD (Niego et al. 2023). After the discovery of cephalosporin C (**4**) (Newton and Abraham 1955) and its semisynthetic derivative cephalotin (**33**), which was marketed in 1964 as the first clinical cephalosporin antibiotic, a whole range of broad-spectrum semisynthetic cephalosporin antibiotics were approved (Lenore et al. 2000). Ceftaroline fosamil (**34**), the last (5^th^) generation cephalosporin with improved selectivity against multi-resistant Gram-positive bacteria, entered the market in 2011 (Critchley et al. 2011; Newman and Cragg 2020). An outstanding example of basic and applied research in pharmacy is the development of the semisynthetic echinocandins rezafungin (**21**), caspofungin (**23**), and micafungin (**25**), which are used as first-line treatment against invasive mycosis (Hüttel 2021). Here, optimized fermentation processes, modification of the product spectrum through mutagenesis, and improved activity and solubility due to chemical modification generated potent antifungal compounds (Hüttel 2021). Designed as a result of lead optimization efforts of joint research among Academia and the pharmaceutical industry around the structure of myriocin (**15**) (Kluepfel et al. 1972), initially discovered as an antifungal metabolite in 1972, fingolimod (**35**) was first synthesized in 1995 with reduced toxicity and improved immunosuppressive activity (Adachi et al. 1995; Volpi et al. 2019). After 15 years of preclinical and clinical studies, **35** has been approved in 2010 for the treatment of multiple sclerosis. By contrast, cyclosporin A (**16**), which is used to prevent rejection of organ transplants (Survase et al. 2011; World Health Organization 2019), is being used as an original natural product that is produced by fermentation of the ascomycete *Tolypocladiuminflatum*.

Applied as the first top-sellers from *Basidiomycota*, the pleuromutilins (**5**, **36**–**37**) are the latest class of antibiotics launched on the market for use in humans. Although pleuromutilin (**5**) was discovered in the early 1950’s (Kavanagh et al. 1951), its semisynthetic derivative retapamulin (**36**) entered the market in 2007 as a new class of antibiotics following a long innovation gap (Daum et al. 2007; Novak 2011). Noteworthily, the semisynthetic pleuromutilin antibiotic lefamulin (**37**) was recently approved by the EMA and is used for systemic treatment of bacterial infections in humans (Veve and Wagner 2018; Newman and Cragg 2020; Mapook et al. 2022). Other prominent fungal metabolites in use are the strobilurins (**26**–**27**) which are now established as one of the most important agents of agrochemical fungicides world-wide (Sauter et al. 1999). Based on mimetic synthesis, there are currently ten major derivatives on the market representing 23–25% of the global sales in the agrochemical sector (Anke 2020).

To date, fungal secondary metabolites continue to be exploited as a source for new drugs. An example for such a compound would be psilocybin (**38**, Kargbo et al. 2020), which is currently in clinical trials, while others were only recently approved. The latter applies to ibrexafungerp (**39**), a semisynthetic derivative of the triterpenoid enfumafungin (**40**) which was approved as orphan medicine (EMA 2021), as well as for the next-generation echinocandin rezafungin (**21**), a structural analog of anidulafungin (EMA 2024; see also Table 1).

**Figure 2.**
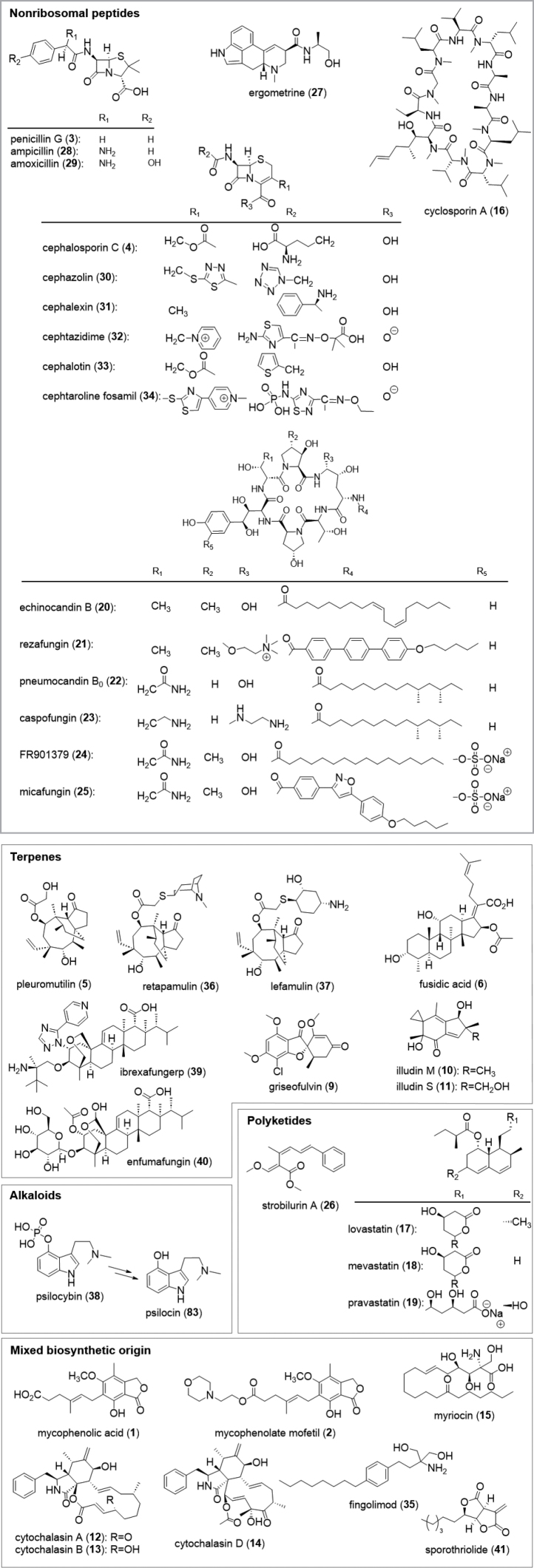
Examples of important natural products together with their semisynthetic analogues from fungi grouped by biosynthetic origin.

Secondary metabolites are derived from central metabolic pathways, analogous to primary metabolites. The secondary metabolism in fungi is mostly encoded by genes organized in BGCs that encode dedicated enzymes to catalyze various reactions known from synthetic and organic chemistry (Keller 2019). Typical examples of natural product classes are i) the PKs, produced from malonyl- and Ac-CoA units formed by polyketide synthases; ii) NRP generated by using amino acids as templates; iii) the terpenoids, produced by terpene synthases and cyclases with isoprene units as basic building blocks, iv) alkaloids, generated from amino acids, and v) combinations thereof, such as meroterpenoids (mero = partial) (Fig. 2). Aside from these biosynthetic pathways, there are some rare ones discovered in fungi, such as the alkyl citrates, exemplified by the antimycotic sporothriolide (**41**) (Tian et al. 2020; Kuhnert et al. 2021). A rising number of secondary metabolites originating from ribosomally synthesized and posttranslationally modified peptides have been described over the last years (Bills and Gloer 2016; Walsh and Tang 2017; Keller 2019; Vogt and Künzler 2019). Examples for important secondary metabolites from fungi together with their semisynthetic analogues, categorized according to their biosynthetic origin, are given in Fig. 2.

Over the last century, natural product discovery has undergone its own process of evolution. Although strategies for the isolation of natural products were less complex, chromatographic technologies relatively limited, and methods for structure elucidation at the very beginning, the early stage of natural product research (1940s–1970s) was very productive (Katz and Baltz 2016; Karwehl and Stadler 2017). Nowadays, significant improvements in analytical techniques, assessment of the potential prolificity of a surveyed strain by genome mining, biological manipulation together with engineering strategies, and microbial culturing have made this laborsome work far more efficient (Atanasov et al. 2021). Hence, it can be expected that continuous technical advancements will further catalyze the description of many more thousands of secondary metabolites, waiting to be characterized also for potential biotechnological applications (for an overview of some remarkable secondary metabolites used in biotechnology, cf. Hyde et al. 2024). In the following, we will give examples of different approaches to evaluate and describe the secondary metabolome of fungi.

## ﻿Fungal-derived natural product discovery – methodologies from the past to the future

### ﻿Reflections of the past – Seeing is believing

**Figure 3.**
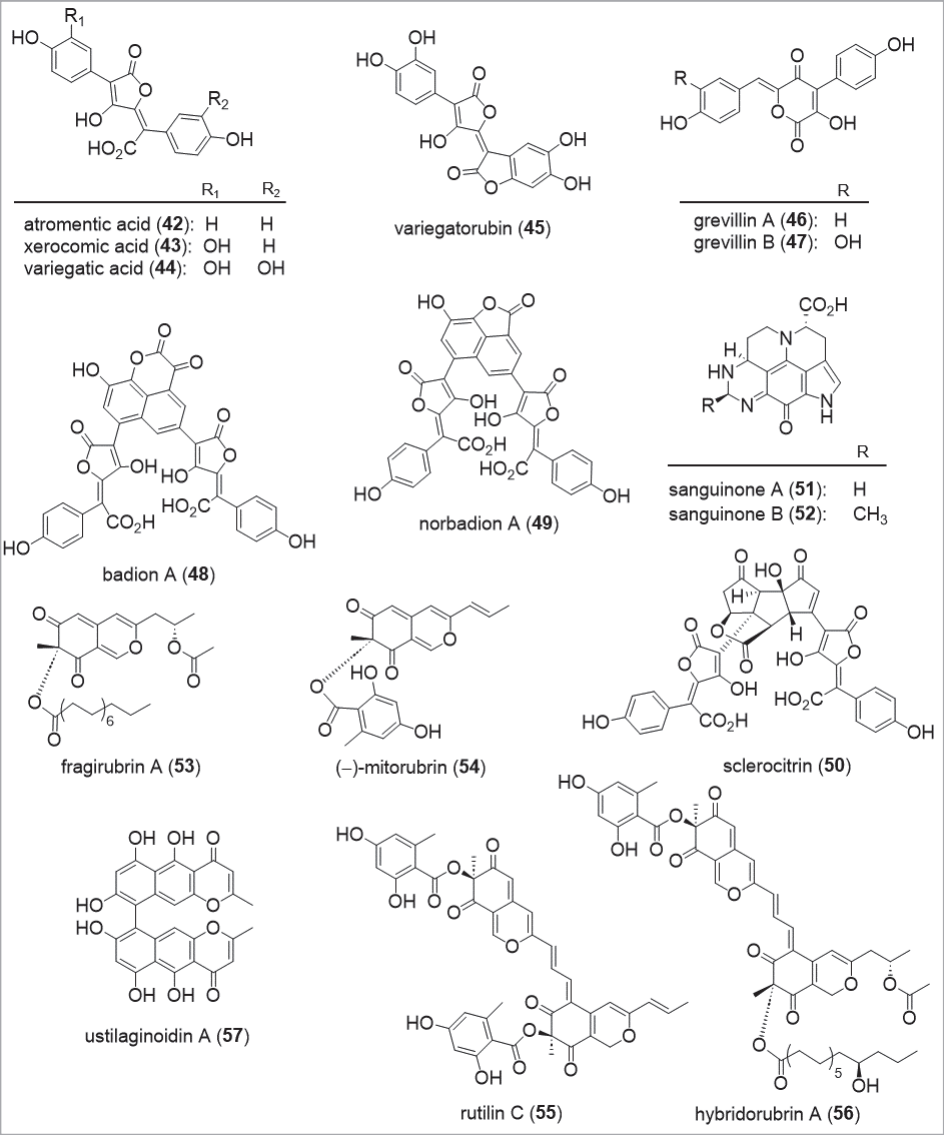
Examples for the structural diversity of pigments from fungi.

Many secondary metabolites were discovered from fungi sparked by the fascination for promising bioactivities, harmful poisons, or colorful pigments. In particular, fungi exhibit a variety of colors and color changes, which attracted the attention of organic chemists, facilitated by the fact that pigments were visible during the separation process. Bright pigments such as the pulvinic acids (**42**–**45**) and the grevillins (**46**–**47**) were already isolated in the 1960s and 1970s from the basidiomata of the *Boletales* (Fig. 3; Gill and Steglich 1987). Due to the advent of sophisticated chromatographic and spectral techniques, many additional, complex and fascinating pigments have since then been discovered. Those include the orange-brown naphtaloid pulvinic acids badione A (**48**) and norbadione A (**49**) from the cap skin of the Bay Bolete (now called *Imleriabadia* or *Xerocomusbadius*) (Steffan and Steglich 1984), the bright yellow triquinanoid pulvinic acid sclerocitrin (**50**) (Winner et al. 2004) from fruiting bodies of *Sclerodermacitrinum*, and the blue colored sanguinones (**51**–**52**) from *Mycenasanguinolenta* (Peters and Spiteller 2007). Besides the intriguing colors of fruiting bodies of *Basidiomycota*, the stromata of *Ascomycota* have been shown to be a prolific source of pigments as well (Caro et al. 2015). During a study on stromatal extracts of *Hypoxylonfragiforme*, 19 complex pigments of the fragirubrin- (**53**), mitorubrin- (**54**), rutilin- (**55**), and hydrorubrin-types (**56**) were isolated, demonstrating the great diversity of azaphilones in *H.fragiforme* (Becker et al. 2021). Notably, archeological dating methods and analytical chemistry suggested the prevalence of these pigments over millennia in fossilized stroma (Surup et al. 2018a).

### ﻿Recent advances – Technical innovations driving modern natural product discovery

At the outset of natural product discovery, NMR spectroscopy was still in its infancy and large amounts of metabolites were needed for basic experiments. For instance, in 1963, a proton NMR spectrum at 60 MHz was performed with amounts of 20–30 mg of the compounds, as Shibata demonstrated for the structure of ustilaginoidin A (**57**) (1963). Structures of unknown metabolites were mostly solved by means of organic synthesis strategies like degradation or derivatization reactions (Beaumont et al. 1968; Steglich et al. 1970), or in tandem with synthesis and NMR spectroscopy. Commonly applied chromatography techniques for the isolation of fungal metabolites comprised column chromatography on NP (e.g. silica gel), or SEC (e.g. Sephadex^TM^LH-20) as well as TLC. Another strategy follows crystallization procedures, as used, for example, for the isolation of pulvinic acid derivatives (**44**–**45**) or anthraquinone derivatives (Madhosingh 1966; Beaumont et al. 1968; Edwards 1977; Besl et al. 1978). Over time, experimental basics for the discovery of new compounds – such as screening, extraction and isolation of pure compounds – remained largely unchanged. Significant advancements, like the integration of hyphenated instruments, the application of AI, the diversification of chromatographic solutions and the increase in sensitivity, have complemented these basics substantially (Table 2; Newman and Cragg 2020). Isolation and structure elucidation of new compounds can now be achieved more rapidly and with decreasing sample amounts, so that even minor constituents of extracts are attracting growing attention in the discovery of fungal metabolites.

**Figure 4.**
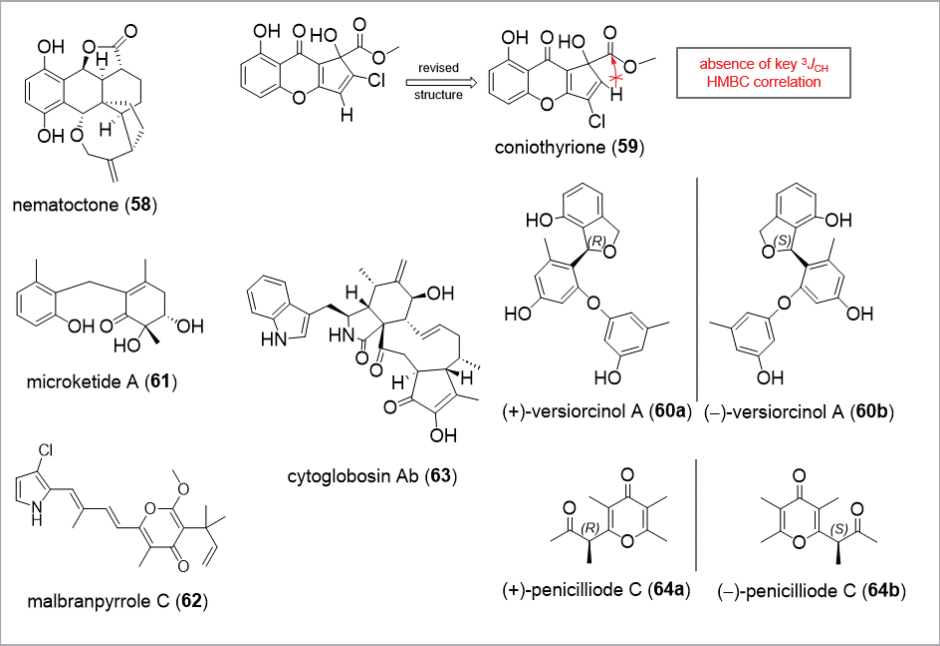
Examples for challenging structures in the discovery of natural products of fungi.

**Table 2.** Technical advances and new strategies in natural product chemistry.

field	development		impact	example
**NMR spectroscopy** ^a^	instrument properties	**high-field NMR** (e.g. 600 MHz, 800 MHz, 900 MHz)	increased sensitivity and quality	**nematoctone** (**58**) from *Hohenbueheliagrisea* (0.6 mg sample amount, 5 mm cryoprobe, shigemi tube, 700 MHz)^b^
		**microprobes** (reduced diameter [1–3 mm; 10–140 µL], shigemi tubes, microcoil flow)	increased sensitivity towards enhanced signal to noise ratio (S/N)	
		**cryogenic probes**		
	sophistica-ted 2DNMR experiments	**homo**- [^1^H-^1^H and ^13^C-^13^C], **heteronuclear** [^1^H-^13^C, ^1^H-^15^N, ^13^C-^15^N] **direct** and **(ultra-)long-range experiments** (**LR-HSQMBC**, **LR-serHSQMC**)	for challenging structures (e.g. low sample amount, highly proton-deficient core structures, weak heteronuclear correlations)	structure revision of **coniothyrione** (**59**, moderate antibacterial) from *Coniothyriumcerealis* (1.2 mg sample amount, 1.7 mm MicroCryo-Probe^TM^, 600 MHz)^c^
	computa-tional tools	**computational modeling of ^1^H, ^13^C chemical shifts** (**h**ierarchical **o**rganization of **s**pherical **e**nvironments [**HOSE**] code algorithms in combination with **m**achine **l**earning methods [**ML**])	assistance in structure elucidation and verification	**(±)-versiorcinols A** (**60a**, **60b**, moderate antibacterial) from *Aspergillusversicolor* (**g**auge **i**ndependent **a**tomic **o**rbital [GIAO], **s**pin-**s**pin **c**oupling **c**onstants [SSCCs])^d^**microketide A** (**61**, antifungal) from *Microsphaeropsis* sp. (GIAO)^e^
		**c**omputer **a**ssisted **s**tructure **e**lucidation (**CASE**) software creating a **m**olecular **c**onnectivity **d**iagram (**MCD**)		
**mass spectrometry** ^f^	instrument properties	**ionization source** (**e**lectro**s**pray **i**onization [**ESI**], **m**atrix-**a**ssisted **l**aser **d**esorption/**i**onization [**MALDI**], **d**esorption **e**lectro**s**pray **i**onization [**DESI**])	increased application range	*in situ* study on fungal metabolites in (co)-cultures (DESI-MS imaging) ^g^
		(**high-resolution**) **mass** a**nalyzer** (**t**ime-**o**f **f**light **m**ass **s**pectrometry [**TOFMS**], **q**uadrupole **m**ass **s**pectrometry [**QMS**], **QTOF**, **ion trap**, **Orbitrap**)	increased sensitivity, speed and quality; for MS/MS applications	quantification of trace levels of **triterpenoids** in *Ganodermalucidum* (UPLC-ESI-HR-QTOF-MRM)^h^
	combined techno-logies	**separation techniques** (**u**ltra **h**igh **p**erformance **l**iquid **c**hromatography [**UHPLC**], **i**on **m**obility **s**pectrometry [**IMS**])	increased resolution and speed of analysis; for HTS applications	screening of ≈13.000 fungal extracts (HTS profiling via UHPLC-MS)^l^
**hyphenated techniques** ^i^	Instru-mentation	**coupling h**igh **p**erformance **l**iquid **c**hromatography (**HPLC**), **NMR**, **IMS**, **c**ircular-**d**icroism (**CD**), or **SPE** (e.g. LC-NMR, LC-IMS, LC-CD, LC-SPE-NMR)	on-line analysis of complex biological matrices (e.g. unstable metabolites)	**malbranpyrrole A** (**62**, cytotoxic) from *Malbrancheasulfurea* (LC-SPE-NMR, photosensitive polyketide)^j^
**chromato-graphy** ^k^	combined techno-logies	**2D-LC techniques**	increased peak capacity, selectivity and resolution; for preventing degradation of unstable compounds	**cytoglobosin Ab** (**63**) from *Chaetomiumglobosum* (preparative MPLC × HPLC system)^m^
	material	**r**eversed **p**hase (**RP**), hydrophilic interaction chromatography (**HILIC**), **core-shell particles**, **chiral stationary phases**	increased resolution	**(±)-penicilliodes C** (**64a**, **64b**) from *Penicillium* sp. (separation on chiral stationary phase)^n^
**comple-mentary approaches** ^o^	computa-tional tools	**dereplication**	wide analyte coverage, increased sensitivity and selectivity; for HTS applications	**oligoisoprenoids** and **styrylpyrones** from *Gymnopilusimperialis* (dereplication via GNPS)^p^ novel **azaphilones** from *Parahypoxylon* spp. (UHPLC-DAD-IM-MS/MS)^q^
		untargeted (MS)-based **metabolomics**		

^a^ (Fukushi 2006; White et al. 2008; Senior et al. 2013; Halabalaki et al. 2014; Sergey et al. 2014; Williamson et al. 2014; Elyashberg 2015; Martin et al. 2015; Andernach et al. 2016; Wolfender et al. 2019; Motiram-Corral et al. 2020; Elyashberg and Argyropoulus 2021); ^b^ (Sandargo et al. 2018); ^c^ (Ondeyka et al. 2007; Martin et al. 2013); ^d^ (Gu et al. 2017); ^e^ (Liu et al. 2020a); ^f^ (Arevalo et al. 2019; Dodds and Baker 2019; Masike et al. 2021); ^g^ (Sica et al. 2014); ^h^ (Kaewnarin et al. 2021); ^i^ (Gebretsadik et al. 2021); ^j^ (Yang et al. 2009); ^k^ (Gritti et al. 2007; DeStefano et al. 2008; Chen et al. 2012; Stoll and Carr 2017; Atri et al. 2019; Zeng et al. 2019; Brandão et al. 2020); ^l^ (Ito et al. 2011); ^m^ (Yan et al. 2016); ^n^ (Wei et al. 2019); ^o^ (Bitzer et al. 2007; Li et al. 2022; Palermo 2023); ^p^ (Caldas et al. 2022); ^q^ (Cedeño-Sanchez et al. 2023).

## ﻿Workflow – from the fungus to the compound

### ﻿Sources of novel metabolites and the importance of taxonomy

**Figure 5.**
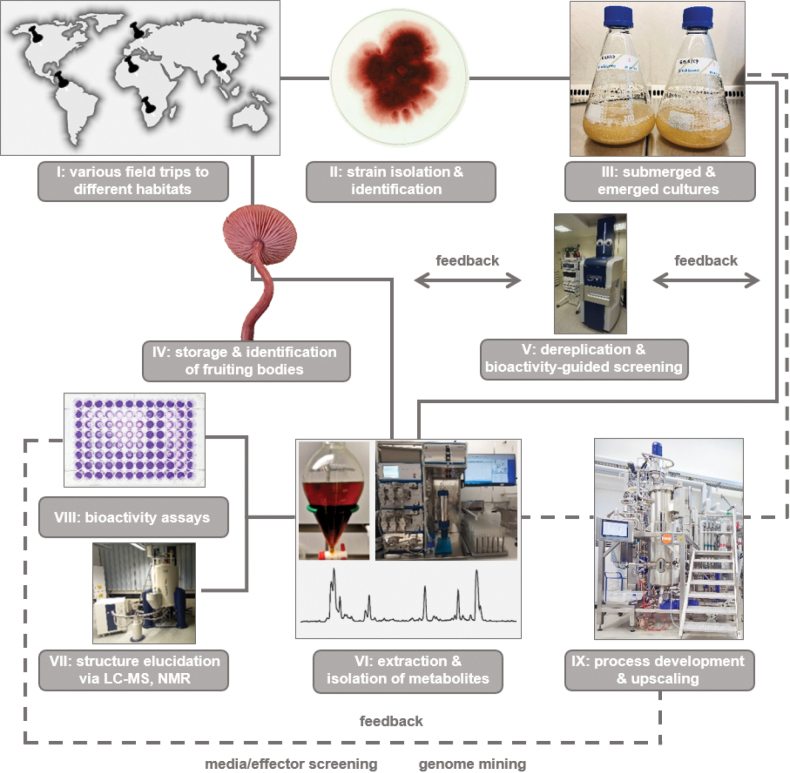
Different strategies for the exploitation of fungal sources (Photos: Lillibeth Chaverra-Muñoz (III); Hedda Schrey (II, IV, VI, VIII); Nina Sandmann (V, VII); Frank Surup (IX).

To study the natural product chemistry of fungi, the biological material for examination must naturally be obtained first. For this purpose, fungal material collected from various geographic or ecological contexts – in accordance to local and global regulatory law –, can be used (step I, Fig. 5). Readily visible fruiting structures (e.g. ascomata and basidiomata of macrofungi) can be collected during field trips and pure cultures isolated from their spores or their mycelial tissue (step II, Fig. 5). Subsequently, they can be cultivated (step III, Fig. 5). Based on micro- or macro-morphological characters and DNA sequence data, a sound determination of a fungus’s taxonomic affinities is essential to ensure the identity of the collected (and isolated) organisms, together with the deposition of vouchers in official biodiversity repositories. The pitfalls of inadequate or inaccurate taxonomic treatments of important secondary metabolite producers can be seen in two independent examples: a) The producer of the cyclodepsipeptide PF1022A, which is semi-synthetically modified to yield the marketed nematicidal drug emodepside, was only tentatively assigned to *Rosellinia* and allies in a patent application by Harder et al. (2011). Only later, Wittstein et al. (2020) unambiguously demonstrated that ascospore-derived isolates of members of the genere *Rosellinia* and *Astrocystis* were indeed able to produce derivatives of the PF1022 family and concurrently resurrected the genus *Dematophora* in the course of a taxonomic study for plant pathogenic *Rosellinia*, that curiously were not able to produce PF1022 derivatives.

Another striking example for the concise identification of important fungal strains that were historically reported to produce bioactive compounds treats the alleged producer of taxol, formerly classified as *Taxomycesandreanae*. The genus *Taxomyces* was originally erected by Strobel et al. (1993) for an endophyte isolated from the taxol-producing Yew tree *Taxusbrevifolia*. The authors postulated that endophytic fungi could produce the plant metabolite and discussed the possibility of horizontal gene transfer between the endophyte and its host. Notably, there is absolutely no evidence for such a phenomenon until today, and it is not plausible because the taxol biosynthesis genes are not even clustered. Many studies followed that claimed the detection of taxol in other endophytic ascomycetes from Yew and even many other plants that do not even produce taxol. None of those studies provided unambigious proof demonstrating that this highly complex molecule can indeed be produced by a fungus. The methodology used was inadequate as no preparative isolation and characterization of natural products by NMR spectroscopy and other salient methods described below was conducted. Later, Heinig et al. (2013) reported that they were unable to find the taxadiene synthase gene, which is essential for taxol biosynthesis, in an Illumina-based genome of the fungal ex-type strain (Heinig et al. 2013). Unfortunately this valuable contribution was largely ignored by principal investigators who drove their students into a dead end, the publication of inconclusive studies – and in particular, reviews that cited those and other inconclusive reviews, did not stop. Based on genome mining for the phylogenetic marker genes, as well as on microscopic studies of the holotype specimen, Cheng et al. (2022) now found out that *Taxomyces* is not even an ascomycete, but a basidiomycete which was assigned to the genus *Ceriporiopsis* (Cheng et al. 2022). This finding made the possibility of horizontal gene transfer even more improbable. Stadler and Kolarik (2024) as well as Gärditz and Cessnick (2024) have critically discussed this phenomenon in the context of scientific integrity and tried to provide a rationale that will hopefully prevent the scientific papermills from spreading nonsense and also direct the supervisors of young scientists to more attractive research goals. While endopytic fungi and many other environmental isolates that represent sterile mycelia could hardly be identified to the genus or species level in the 1990s, this has now changed with the advent of molecular phylogeny and genomics. Even non-specialists such as natural product chemists are increasingly resorting to molecular data for characterization of their producer strains, however, they often only use ITS nrDNA, which does not necessarily yield conclusive results on the identity of their strains. It is hence strongly recommended for non-specialists working with fungi as sources for biologically active compounds to carefully read the recommendations by Raja et al. (2017) and to act accordingly. Ideally, interdisciplinary collaborations with mycological taxonomists would be of profound interest for both fields, natural product research and taxonomy alike.

**Figure 6.**
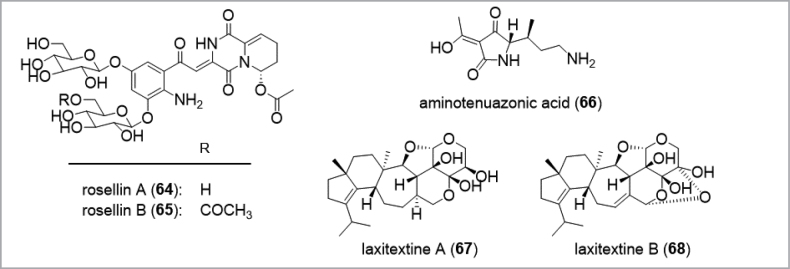
Chemical structures of the rosellins A and B (**64**–**65**), aminotenuazonic acid (**66**), and the laxitextines **A** and **B** (**67**–**68**).

Since the production of secondary metabolites often differs between fruiting bodies harvested in nature and cultured vegetative mycelia in the lab, various strategies have been established to aquire novel compounds from fungal sources. Fruiting bodies, on the one hand, are often only available in limited quantities owing to their short appearance during the mushroom season. Moreover, a holistic chemical characterization of small-sized fruiting bodies can be very challenging, especially if they belong to rare taxa, due to the fact that often more than 50 g (fresh weight) are needed, as exemplified for the isolation of the red diketopiperazine alkaloids rosellins A and B (**64–65**; Fig. 6) from *Mycenarosella*, a tiny mushroom with a cap diameter of only 1–2 cm (Fig. 13, Lohmann et al. 2018). Therefore, an important point to consider is the storage of organic material for subsequent isolation of secondary metabolites, as well as their treatment (e.g. fresh, dried or frozen; Himstedt et al. 2020; step IV, Fig. 5). In contrast, fresh fruiting bodies have often been used for injuring experiments. As a consequence to physical injury of the fungal tissue, wound-activated chemical responses can elicit different secondary metabolites compared to intact fruiting bodies in the framework of a chemico-ecological adaptation strategy (Himstedt et al. 2020). Other fields of use for fresh fungal material are feeding experiments of living fruiting bodies in their natural environment with ^13^C- or ^14^N-labeled precursor molecules for the investigation of biosynthetic pathways. A successful example for monitoring hypothetical pathways is the incorporation of [1,2-^13^C_2_]-acetate during the biosynthesis of aminotenuazonic acid (**66**) in fruiting bodies of *Laccariabicolor*, a 3-acyltetramic acid derivative which might be derived from (2*S*,3*S*)-3-methylornithine and acetoacetyl-CoA (Schrey et al. 2019a).

Mycelial cultures, on the other hand, can easily be expanded for experiments after successful isolation of the pure strains on culture plates. Transferring the organisms to submerged or solid cultures are standard procedures to induce the production of secondary metabolites (step III, Fig. 5). Different media compositions and fermentation conditions can be evaluated in small-scale screening experiments accompanied by analytical methods to evaluate chemical diversity in crude extracts and to designate worthwhile targets for chemical isolation and characterization (step V, Fig. 5). Dereplication – the systematic comparison of spectroscopic data for distinct components of a complex extract with the literature or databases to avoid the isolation of undesired or known secondary metabolites – constitutes an early-stage pre-selection method and can act as a major timesaver (Bitzer et al. 2007; Nielsen et al. 2011; Stadler et al. 2014; Gaudêncio and Pereira 2015; Nielsen and Larson 2015; Wolfender et al. 2019). Dereplication is often done by UHPLC, especially in high-throughput screening scenarios, coupled by DAD and HRMS and HRMS/(MS)^n^ in combination with chemical structure database searches, for example using CAS SciFinder (ca. 183 million compounds), PubChem (ca. 110 million compounds), ChEMBL (ca. 2.1 million compounds), or Dictionary of Natural Products (ca. 328.000 natural compounds). Bioactivity-guided fractionation using a phenotypic screening approach is typically used to evaluate crude extracts, as exemplified by the discovery of the laxitextines (**67**–**68**) from cultures of the basidiomycete *Laxitextumincrustatum* (Mudalungu et al. 2015).

**Figure 7.**
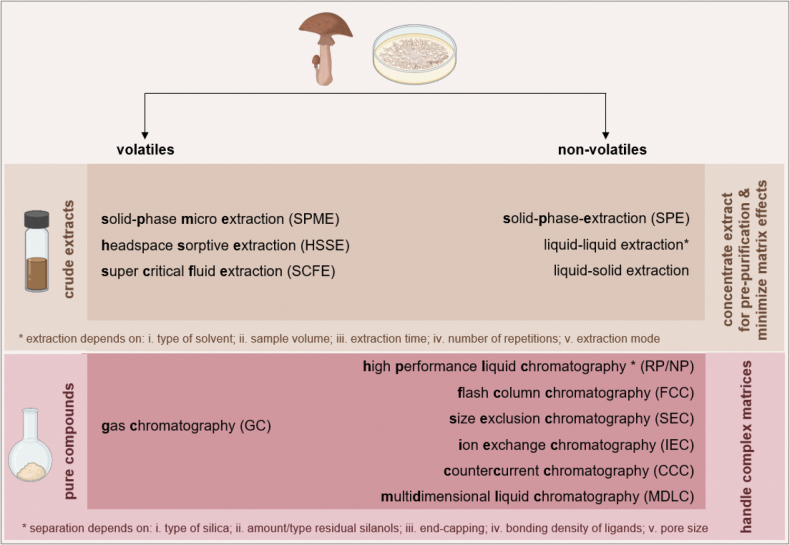
Techniques and chromatographic strategies for isolation of natural products from fungi. Prepared using biorender.com.

Increasing the amount of (crude) extract material by repeating a fermentation in multiple batches or increasing culture volume may be necessary to allow the subsequent isolation of sufficient amounts of pure compound for structure elucidation and broad biological characterization. After separation of biomass and supernatant (only necessary in case of submerged cultures), a variety of extraction techniques and chromatographic strategies (step VI, Fig. 5), elaborately discussed in several reviews (Pucci et al. 2009; Latif and Sarker 2012; Bucar et al. 2013; Marlot and Faure 2017; Sahu et al. 2018; Zuvela et al. 2019; Brandão et al. 2020; Kim and Marriott 2021), are available and summarized in Fig. 7.

### ﻿Structure elucidation of novel metabolites and screening libraries

**Figure 8.**
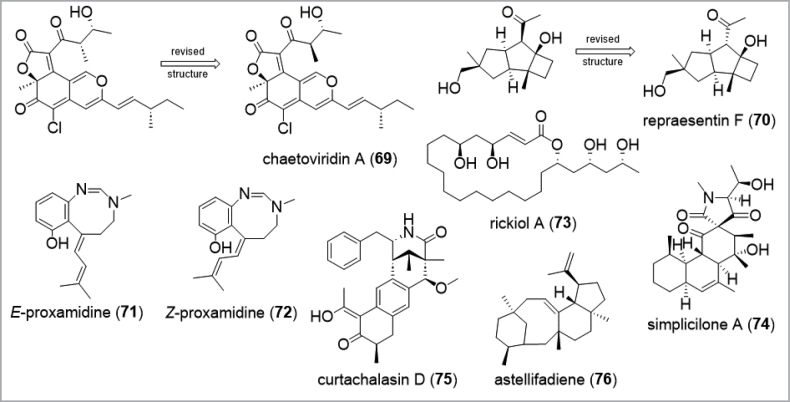
Examples for molecules where it proved challenging to establish the absolute configuration.

After isolation of the pure compounds, their chemical structure can be determined by 1D and 2DNMR spectroscopy and HR-MS experiments (step VII, Fig. 5). Practical strategies for the structure elucidation of small molecules have thoroughly been reviewed (e.g. Kwan and Huang 2008; Breton and Reynolds 2013; Reynolds and Mezzola 2015) and even published in detail in book articles (e.g. Mangoni 2012; Linington et al. 2015). The stereochemical determination of chiral molecules is still a major concern in drug discovery because stereoisomers can considerably differ in potency, toxicity, and behavior (pharmacodynamics). Assigning the absolute configuration can be one of the most challenging tasks in structure elucidation even though a variety of methods have been established. Certainly, total synthesis followed by comparison of the analytical and chiroptical data of the natural and synthetic product (Schrey et al. 2019a), or X-ray crystallography (Mechlinski et al. 1970) are the gold standard to determine the absolute configuration. Notably, numerous examples for structure revisions via total syntheses have been reported, as demonstrated for strobilurin A (**26**) (Anke et al. 1984), the azaphilone chaetoviridin A (**69**) from *Chaetomium* spp. (Makrerougras et al. 2017; Fig. 8), the protoilludane type sesquiterpenoid repraesentin F (**70**) from basidiomes of *Lactariusrepraesentaneus* (Ferrer and Echavarren 2018), or a harziane diterpenoid from *Trichodermaatroviride* (Hönig and Carreira 2020). However, in many cases, natural products are very difficult or even impossible to synthesize in a cost efficient manner and in larger quantities due to their complex structures and the number of chiral centers. Hence, not all of these compounds are applicable for synthesis or suitable for crystallization. As discussed before, during the last decades, technological progress has improved NMR spectroscopy to enable its use as a powerful tool for the stereochemical determination of chiral molecules. Dipolar coupling analysis (NOESY and ROESY) in conjunction with ^1^H-^1^H scalar couplings are the preferred methods for the stereochemical elucidation of cyclic molecules, recently used for the conformation of the eight-membered heterocycles *E*/*Z*-proxamidines (**71**–**72**) (Schrey and Spiteller 2019). In contrast, acyclic and macrocyclic molecules contain carbon chains with higher flexibility allowing multiple slowly interconverting rotamers to be present in the NMR spectrum. To solve these problems of assigning the relative configuration, *J*-based configurational analysis (JBCA, known as ‘Murata’s method’) has been implemented in structure elucidation with great success (Matsumori et al. 1999). This method considers ^3^*J*_H-H_ and ^2,3^*J*_H,C_ coupling constants to assign anti or gauche relationships of vicinally substituted chains, successfully applied for determination of the relative configuration of e.g. rickiol A (**73**) (Surup et al. 2018b) and simplicilone A (**74**) (Anoumedem et al. 2020). The ^1^H-^1^H and ^1^H-^13^C coupling constants are typically measured indirectly through a combination of NMR experiments (Surup et al. 2018b; Anoumedem et al. 2020). Aside from residual dipolar coupling analysis, which was used to assign the relative configuration of curtachalasin D (**75**) from Xylariacf.curta (Wang et al. 2019c), the concept of a universal NMR database approach from Kishi’s group is worth mentioning for stereochemical assignment of polyketides (Kobayashi et al. 1999; Lee et al. 1999; Kobayashi et al. 2000a; Kobayashi et al. 2000b; Kobayashi et al. 2001). Based on systematic observations of differences in ^1^H NMR and ^13^C NMR chemical shifts of synthesized highly functionalized and acyclic model compounds, numerous NMR data-sets of stereoclusters are available for comparison and determination of natural products with unknown stereochemistry containing the respective structural motif (Matsumori and Murata 2017; Ma et al. 2020).

For establishing the absolute configuration, derivatisation reactions as well as shift reagents (Jiménez-Romeo et al. 2010) have often been used. Known as Mosher’s method, i.e. derivatization with MTPA followed by analysis of NMR chemical shift differences of the MTPA esters, represents the most widely used tool for the assignment of secondary hydroxyl functions (Dale et al. 1969). Problems can arise when the molecule features multiple functional groups, such as alcohol or amino groups, leading to several reaction products. For the stereochemical assignment of amino acid constituent units, hydrolytic cleavage and derivatization of the resulting amino acid with a chiral reagent followed by subsequent comparison of the diastereomer with authentic (synthetic) samples of known configuration on achiral column materials via HPLC or GC deliver the absolute configuration of chiral compounds. This strategy has been established as Marfey’s method and other methods derived thereof (Marfey 1984; Vijayasarathy et al. 2016; Schrey et al. 2019b; Harms et al. 2021). Aside from NMR chiral solvating agents (Pedras et al. 2005), the crystalline sponge method can be implemented in absolute configurational analysis of complex novel metabolites, as demonstrated for the determination of the absolute configuration of the sesterterpene astellifadiene (**76**) from “*Emericella*” (correctly referable to *Aspergillus* in current One-Fungus-One-Name-based taxonomy!) *variecolor*, which was heterologously expressed in *Aspergillus* (*flavus* var.) *oryzae* (Matsuda et al. 2016). Each method has its specific limitations and it is often necessary to combine two or more methods. For instance, the stereochemical analysis of rickiol A (**73**), JBCA in conjunction with Kishi’s method was applied to establish the relative configuration, followed by Mosher’s method for the absolute configuration (Surup et al. 2018b). On a last note, we wish to comment on the usage of ECD, which is based on the comparison of experimental ECD and calculated ECD spectra, as it has become a sought-after tool for establishing the absolute stereochemistry of natural products (Li et al. 2010; Superchi et al. 2018). ECD calculations, which to our experience are often requested during the review process, should be used more carefully, as the corresponding calculations are often very time-consuming and may occupy a supercomputer for several months for one stereoisomer, especially for complex natural products with many stereocenters. This specifically applies for molecules that are already defined by X-ray crystal structures or their biosynthesis, to prevent the waste of unnecessary resources. Calculation-based methods have even led to incorrect assumptions in the past (Schmiedel et al. 2018).

Irrespective of isolation strategy or compound priorization, it is opportune to collect isolated substances and extracts in screening libraries both to access their biotechnological potential and to help with dereplication at the beginning of isolation campaigns (Stadler and Hellwig 2004; Bitzer et al. 2007; Barnes et al. 2016). While the search for novel carbon skeletons is rewarding, as the chance of finding novel bioactivities or targets is higher, isolating and screening highly similar compounds (and knowing by which taxonomic groups they are produced) can help with establishing a structure-activity relationship, which is useful information for later lead optimization by medicinal chemists (Stadler and Hellwig 2004; Bauer and Brönstrup 2014; Guo 2017; Silva and Emery 2018; Atanasov et al. 2021).

### ﻿Bioreactor process development for promising candidates

Sufficient quantities for lead structure development and clinical trials are needed when evaluating the suitability of a compound to serve as a drug lead. To increase product yields as well as production titers, fermentation volume can simply be increased (e.g. 15 L, 350 L bioreactors; step IX, Fig. 5). In order to ensure stable production titers and yield, the fermentation process needs to be developed priorly, involving optimization of culture media, process conditions, and process parameters, together with complex analytical and preparative chromatography. A thriving example for a successful upscale within the *Basidiomycota* was the optimization of the production process of illudin M (**10**) produced by *Omphalotusnidiformis*. Development of a scalable and low-cost downstream process together with a robust transfer of gram quantities from shake flask to stirred tank paved the way for its potential application as precursor for semisynthetic anticancer agents (Chaverra-Muñoz et al. 2022; Chaverra-Muñoz and Hüttel 2022).

In contrast to in-culture produced compounds, a substantial amount of promising compounds are exclusively isolated from fruiting bodies. Scale up of those compounds would require extensive amplification of the biomass of a given producer organism and tends to be especially difficult or even impossible, given that the vast majority of fruiting bodies cannot be grown, or induced, artificially due to a variety of reasons. One, apart from the many biological reasons, is simply related to the Cost of Goods as production for industrial applications would often not be feasible. Other economic problems are associated with culture media difficult to scale, such as solid-phase media, and low production titres for which yield optimization using the aforementioned methods failed so-far, preventing their industrial applicability. In these cases, bioengineering tools are available, for which the antibiotic pleuromutilin (**5**), a tricyclic diterpene, is a successful example. Here, the recently discovered biosynthetic gene cluster comprising seven genes was heterologously expressed in *Aspergillusoryzae*. The successful reconstruction in *A.* (*flavus* var.) *oryzae* increased the production of pleuromutilin (**5**) significantly to more than 20-fold compared to the wild-type producing organism, *Clitopiluspasseckerianus*, which turned out to be crucial for its development as a commercial drug (Bailey et al. 2016).

On another note, fermentation, or even total biosynthesis might constitute powerful methods for the production of desired drug candidates, especially when considering to sustainably make use of waste streams in the frame work of a circular economy and in general, environmentally friendly conditions (Cox 2024). However, these ideas have to be brought to fruition first and until then, traditional strategies, like for example chemical total synthesis or synthesis inspired by biosynthesis in the frame of biomimetic reactions can do the job, as is the case, for example, for the production of the strobilurins or the statins (**17**, **19**).

## ﻿Biological aspects

### ﻿Classical fermentation experiments – One strain, many compounds

Before the vast hidden chemical diversity of microorganisms and fungi became apparent using modern molecular biological and bioinformatic tools, the effect of even small changes in the composition of culture media and cultivation conditions was already noted and documented by empirical evidence. This includes the influence of culture aeration during fermentation (aspinolides, aspinonenes and aspyrones; Fuchser and Zeeck 1996) as well as the addition of supplements such as sodium bromide (hexacyclinic acid, Höfs et al. 2000). Observations for the variability of the secondary metabolite production under standardized laboratory conditions have been unified under the OSMAC hypothesis (see Bode et al. 2002). The hypothesis follows the idea that changes in environmental factors serve as impulses to the metabolic and ultimately the biosynthetic program of the surveyed organism to adapt to its current surrounding, as is programmed by the genetic code. For bioprocess development, several scenarios can be tested in dependency of the technical and experimental setup: impact of pH control, shear stress, process temperature, and oxygen supply (controlled by biotechnological machinery). Media components, especially considering its source (C/N ratio), can be crucial, but can also deliver important precursors (Rinkel and Dickschat 2015), effectors (glucose catabolite repression in *A.flavus*; Fasoyin et al. 2018), and inductors (glycerol in cephalosporin production; Shin et al. 2010). Even the culture morphology, which can be controlled by altering the growing environment with inert minerals (Antecka et al. 2016; Veiter et al. 2018) or the transfer of the process to solid growth media instead of liquid cultivation (Son et al. 2018), as well as light stress, can have meaningful influence on the production of secondary metabolites. Many fungi are able to sense light with the help of photosensitive proteins (Fuller et al. 2014; Lawrinowitz et al. 2022), which can contribute to the pigmentation of a fungal culture, an important causal factor shown to accompany growth stage progression (Yu and Fischer 2019). While these traditional variations in growth conditions lead to the discovery of several thousands of natural products and is usually among the first strategies to chemically characterize new species of interest, the number of found secondary metabolites is usually far lower than the number of predictable biosynthetic gene clusters, which remain ‘silent’ in standardized fermentation experiments. Strategies to activate these silence clusters include the co-cultivation with potential competitors or potential biosynthetical precursors (Fischer et al. 2016; Zhang and Elliot 2019). However, such strategies have limited use for industrial applications, as scale-up of such processes is not easy. For example, scale-up of a dual culture system is often already problematic when attempting to transfer production from agar plates to shake flasks.

### ﻿Regulation and appearance of secondary metabolites encoding gene clusters in fungi

Secondary metabolites are products of an orchestrated genetic machinery, which mostly, but not necessarily, occur clustered in a pathway dependent manner (Rokas et al. 2018). Physical transcriptional access to these clusters is regulated by chromatin packaging (euchromatin, active; hetereochromatin, inactive), which itself is governed by a number of post-transcriptional (epigenetic) modifications of the associated histones forming the nucleosome (Gacek and Strauss 2012). Transcriptional regulation of BGCs can either be conceived to act in a local, cluster-specific way or globally, by e.g. affecting chromatin packaging (such as the previously discussed abiotic factors). Some of the best studied examples stem from the work on *Aspergillus*, *Penicillium* and *Fusarium*, probably due to their implications on human health as pathogens (e. g. *A.fumigatus*); being important plant pathogens (e.g. *Fusarium* spp.); or due to their biotechnological importance (e.g. *P.chrysogenum*). An example for global transcriptional regulators are members of the velvet-complex (VelB/VelA/LaeA) in *A.fumigatus* and *A.nidulans*, which have been described to govern fungal development, including its secondary metabolism (Perrin et al. 2007; Bayram et al. 2008). Another global regulator of fungal behavior was found in transcriptional studies of *F.graminearum* with FgStuA, a transcriptional factor exhibiting a highly conserved APSES amino acid sequence domain (see Zhao et al. 2015). Targeted deletion diminished transcription of well-known secondary metabolite encoding genes of the trichothecene and aurofusarin families and concurrently lead to loss of spore production, indicating a link of developmental stage and secondary metabolism (Lysøe et al. 2011). A transcription factor involved in oxidative stress response of *A.parasiticus*, AtfB was shown to bind to sequence motifs involved in aflatoxin biosynthesis (Roze et al. 2011). In *Trichodermareesei*, the deletion of the repressor of xylan degradation Xpp1 led to an increase of detectable transcripts predicted to be involved in polyketide biosynthesis (Derntl et al. 2017). In *Beauveriabassiana*, the transcription factor PacC, previously shown to steer responses to changes in the surrounding pH value (Tilburn et al. 1995), is involved in the regulation of bassianolone B production (Luo et al. 2017). In these examples, genetic targeting enabled the investigation of how environmental cues govern the expression of genes *via* transcription factors at the top of the hierarchy, ultimately steering which genes are activatable at a given moment and which not. The mode of transcriptional regulation for the vast majority of BGCs is, however, unknown and the clusters products hence inaccessible in standard laboratory conditions. Whether this is entirely due to the lack of specific signals leading to unfavorable chromatin packaging, their deactivation in the absence (or presence) of specific signals effected by biotic or abiotic factors, or even due to them being non-functional, is equally unclear (Gacek and Strauss 2012; Rokas et al. 2018; Collemare and Seidl 2019; Rokas et al. 2020). Collemare and Seidl (2019) argued that the field focused only on a handful of well-studied post translational modifications, such as histone acetylation and deacetylation (which can also be manipulated by using chemical inhibitors) affecting chromatin packaging and that more complex, multi-level regulatory mechanisms may be at play. It will be interesting to explore these potentially complex regulatory modes, which might open more directed ways of designing empirical studies to evaluate a strains productive capacities. Key to this will be broad genomical and genetical accessibility of fungal strains. Until then, other approaches are necessary to activate and elucidate the products of cryptic, untranscribed gene clusters, such as expression of the target cluster in a heterologous host, which will be further discussed in section 1.5. For additional information on the evolutionary origin of biosynthetic gene cluster formation and its regulation, we want to direct the inclined reader to other recent reviews covering the available published scientific literature (Collemare and Seidl 2019; Rokas et al. 2020).

### ﻿Ecological context of secondary metabolites produced by fungi

Fungi co-exist with scores of other organisms in their natural habitats. They need to deal with competitors, predators, and UV radiation for sufficient nutrition, space, and survival (Keller 2019). For millions of years during the process of evolution, fungi have developed strategies to secure their survival in highly competitive ecological niches.

**Figure 9.**
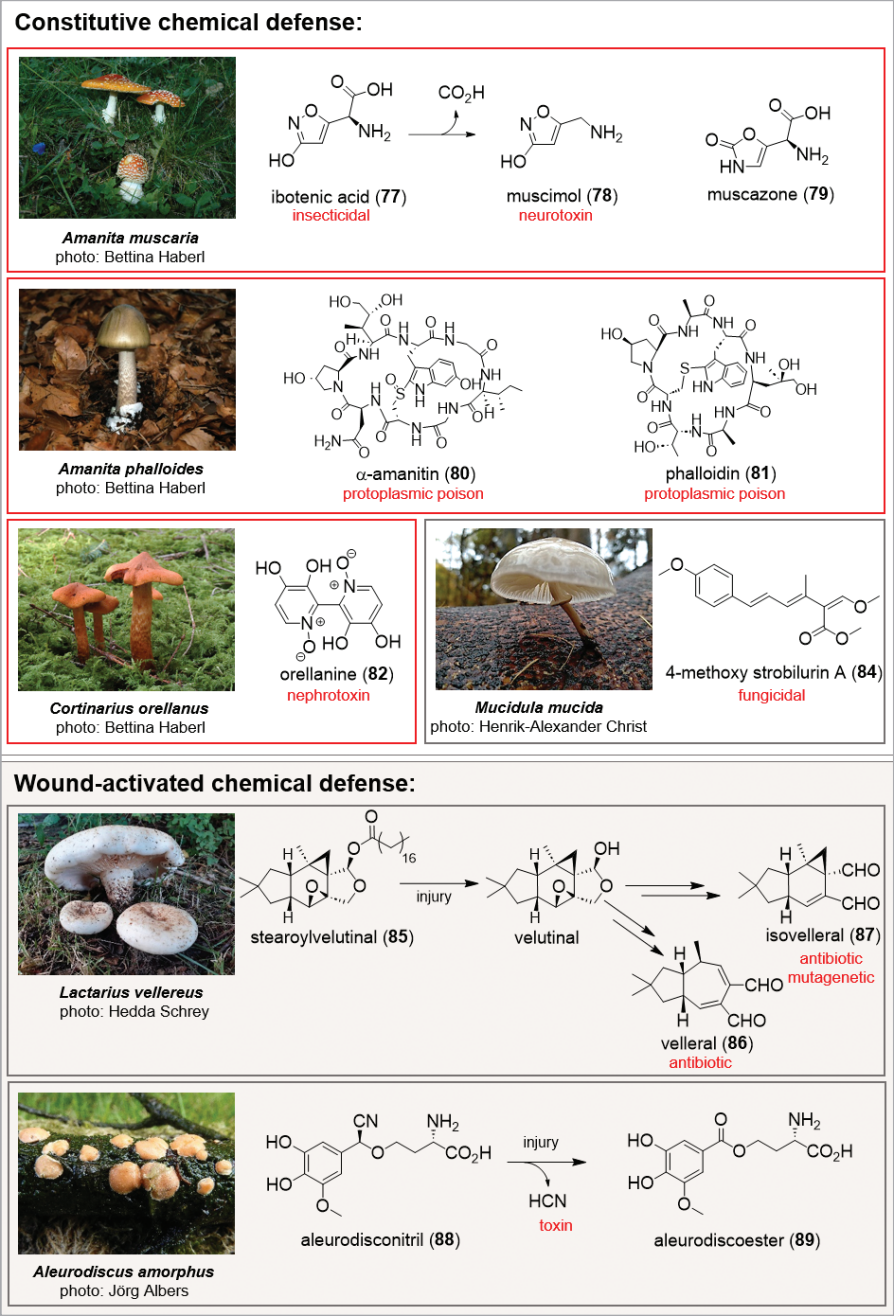
Examples for chemical defense strategies (red outlined examples caused and causes serious mushroom poisoning in the past and present).

Because of their immobility, they have developed a multitude of chemical defense strategies to defend themselves against fungi, bacteria, springtails, nematodes, insects, and other fungivores. The ecological roles of secondary metabolites from fungi have been elaborately reviewed (Rohlfs and Churchill 2011; Spiteller 2015; Macheleidt 2016; Keller 2019). In analogy to plant-herbivore interactions, fungi employ various strategies: constitutive chemical defense, wound-activated defense, and induced chemical defense (Spiteller 2008). Chemical defense agents can be toxic constituents, or bitter and pungent compounds with highly functionalised carbon skeletons equipped with chirality and biological activity (Fig. 9). To determine an ecological function of a secondary metabolite or to understand and investigate its mode of action can be a daunting task. In some cases it is possible to deduce the function from a strong biological activity, for instance ibotenic acid (**77**), an active constituent of the fly agaric (*Amanitamuscaria*) with its insecticidal activity or the antifungal 4-methoxy strobilurin A (**84**), isolated from *Mucidulamucida* (syn. *Oudemansiellamucida*) (Vondráček et al. 1983). Other examples constitute muscimol (**78**), and muscazone (**79**), which act as gamma-aminobutyric acid receptor affecting the central nervous system (Lee et al. 2018; Rivera-Illanes and Recabarren-Garjardo 2024). Additional important toxic components are α-amanitin (**80**) and phalloidin (**81**) from *A.phalloides* or the nephrotoxine orellanine (**82**), present in the fruiting bodies of *Cortinariusorellanus* and *C.rubellus* causing serious mushroom poisoning, as well as the psychotropic psilocin (**83**) from many *Psilocybe* species. However, there are others where extrapolation from effects on humans and a potential ecological function is not trivial. (Antkowiak and Gessner 1979; Fricke et al. 2017). In case of the psilocybin topic, for example, despite decades of research about biosynthetic pathways, chemical mechanisms, therapeutic potential, or large-scale production, the fundamental question regarding its precise ecological function still remains unsolved (Lenz et al. 2020). Considering the energetic efforts to synthesize and accumulate secondary metabolites, there must be a strong benefit for the fungal organism to justify the production of these highly complex molecules.

A striking example for wound activated defense is the enzymatic conversion of the biologically inactive precursor stearoylvelutinal (**85**) into the sesquiterpenoids velleral (**86**) and isovelleral (**87**) from *Lactariusvellereus* as a response to injury (Sterner et al. 1985). In addition to their pungent taste, the dialdehydes **86** and **87** exhibit broad spectrum activity including mutagenic activities for isovelleral (**87**) (Anke and Sterner 1991). Similarly, the enzymatic oxidation of the cyanohydrin ether aleurodisconitril (**88**) to the aleurodiscoester (**89**) probably causes the release of hydrocyanic acid to protect the fruiting bodies of the crust fungus *Aleurodiscusamorphus* against feeding predators (Kindler and Spiteller 2007).

As recently shown for *Mycenarosea*, interactions involving chemical defense between ‘prey’ and predators can be highly sophisticated and complex. Using formaldehyde (**90**) in a constitutive defense mechanism against *Spinellusfusiger*, *M.rosea* is able to protect the fruiting bodies – to some degree – from infestation with this mycoparasite (Himstedt et al. 2020). On the other hand, *S.fusiger* is producing large quantities of gallic acid (**91**) as a counterdefense agent, which reacts with amino acids and formaldehyde to Mannich adducts to detoxify the formaldehyde (**90**).

Further examples for the production of secondary metabolites as antimicrobial weapons are the antifungal strobilurins (**26**, **84**) (Anke 1995), the anti-staphylococcal calopins, such as 8-deacetylcyclocalopin B (**92**) from *Caloboletusradicans* (Tareq et al. 2018), or the nematicidal laccanthrilic acid B (**93**) from several *Laccaria* species (Schrey et al. 2019b). Most of these studies are based on the evaluation of the compound against a panel of bacteria and fungi using concentrations that are matching the ecological concentrations. Noteworthy, physiologically relevant concentrations were shown to act as an interspecies signal rather than a toxin as reported in a study examining dose-dependent effects of phenazine-derived metabolites in co-culture biofilms of *Pseudomonasaeruginosa* and *Aspergillusfumigatus* (Zheng et al. 2015). While high concentrations of the antimycotics were toxic for the fungus, moderate concentrations affected fungal sporulation and development via oxidative stress regulation.

**Figure 10.**
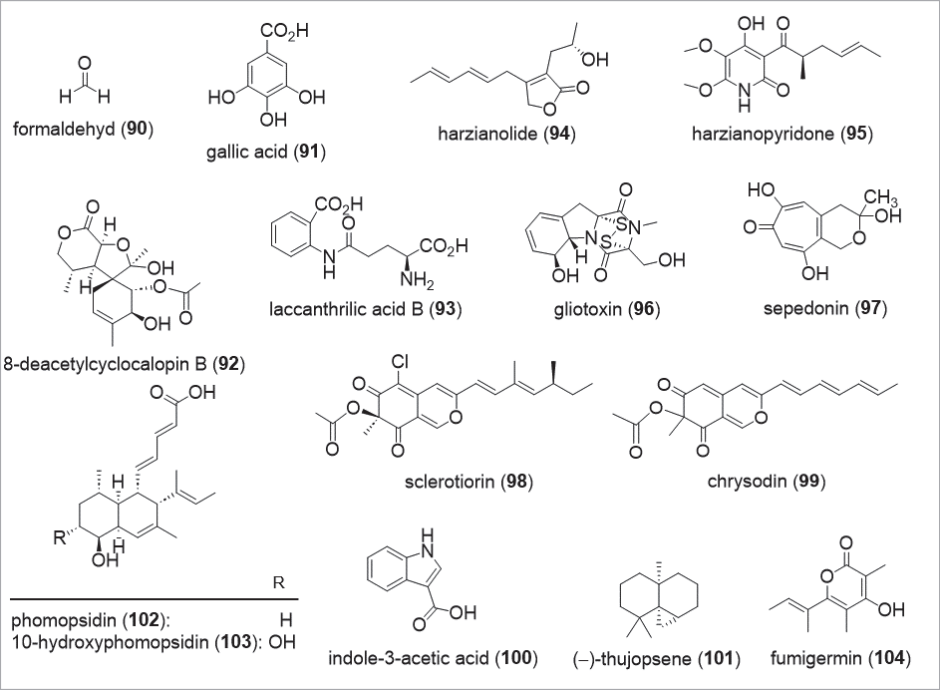
Further examples of the production of secondary metabolites as microbial weapons.

Beside chemical defense mechanisms, fungi are creative artists in establishing symbiotic interactions or conquering habitats by actively attacking other fungi, plants, or insects (Spiteller 2015). For the latter, based on their pathogenic or parasitic lifestyle, these fungi often use cell wall decaying enzymes to infect the host together with toxic compounds to degrade or to handle its chemical defense. Well-investigated examples of the correlation of chemistry and ecological function of secondary metabolites are fungi of the genus *Trichoderma*, commonly encountered as mycoparasites and endophytes, producing different antibiotics such as harzianolide (**94**), harzianopyridone (**95**), trichothecenes, peptaibols or gliotoxin (**96**) (Brian and Hemming 1945; Bell et al. 1958; Dickinson et al. 1989; Cai et al. 2013; Proctor et al. 2018; Marik et al. 2019) and *Sepedoniumchrysospermum*, a necrotrophic mycoparasite that infects the fruiting bodies of *Boletaceae*, producing sepedonin (**97**), (-)-sclerotiorin (**98**), and (-)-chrysodin (**99**) (Wright et al. 1970; Closse and Hauser 1973).

In contrast to this predatory behavior, fungi also frequently form mutualistic relations with various organisms. For ectomycorrhizal associations between plants and *Basidiomycota* in particular, the mycorrhization of the roots is essential for the survival of approximately 6000 species in 145 genera of land plants (Hyde et al. 2019). Despite the fact that ectomycorrhizal associations have been acknowledged for more than one hundred years, little is known about the chemistry, particularly the signaling molecules that initiate mycorrhizal formation, regulation of the nutrient cycle, and interaction with other organisms such as soil bacteria and fungal endophytes (Spiteller 2015). During the pre-colonization phase of the ectomycorrhizal formation, lateral root development is stimulated through non-host specific volatile organic compounds (VOC) acting as chemical messengers to achieve a recognition of both partners (Felten et al. 2009; Ditengou et al. 2015). In case of the basidiomycete *Laccariabicolor*, the phytohormone indole-3-acetic acid (**100**), considered to be one of the main drivers for root formation, could be observed alongside with sesquiterpenes, such as (-)-thujopsene (**101**) (Ek et al. 1983; Ditengou et al. 2015).

Other symbiotic interactions are those of plants and endophytic fungi, where the fungal organism lives inside plant tissue and is part of the microbial community without causing negative effects to the host (Porras-Alfaro and Bayman 2011; Raimi and Adeleke 2021). In this mutualistic relationship, the main paradigm in regard to secondary metabolite research is depicted by the endophytic fungus producing potent bioactive secondary metabolites for plant protection. This assumption is the key principle for the and development of endophytes for biocontrol to protect plants against pathogens. A recently reported and discussed, promising example might be the use of *Hypoxylonrubiginosum* and related taxa against the ‘Ash Dieback’, a chronic disease of the European ash (Halecker et al. 2020; Pourmoghaddam et al. 2020). Producing the antifungal compound phomopsidin (**102**) and its derivative (**103**) in the presence of the pathogen, *Hypoxylonrubiginosum* species could contribute to fencing growth of the invasive *Hymenoscyphusfraxineus* that compromises European forestry.

Continuous development of Omics associated technology is aiding this line of research. Defined as a nonselective, comprehensive, and rapid analytical tool, metabolomics have accelerated modern approaches in chemical ecology and in discovery of novel bioactive metabolites. Based on metabolic profiling of complex biological matrices, metabolome analyzes allow laying focus on intra- and interspecies interactions via hyphenated LC-MS and LC-NMR applications for identification and quantification of metabolites. In the field of comparative metabolomics, chemical profiles are evaluated under different conditions (e.g. an axenic culture versus a stimulated culture) to uncover differences. A promising example of this comparative approach is the discovery of fumigermin (**104**), a novel germination inhibitor of *Streptomycesrapamycinicus* (Stroe et al. 2020). To identify differences as a consequence of fungal-bacteria interactions, the metabolomes of monocultures and co-cultures of *Aspergillusfumigatus* and *S.rapamycinicus* were profiled. This revealed the presence of large amounts of **104** in bacterial-fungal co-cultures, while the axenic fungal culture contained fumigermin (**104**) only in traces. Therefore it can be concluded that an unknown mediator associated with *S.rapamycinicus* triggered activation of the weakly expressed biosynthetic gene cluster of **104** in *A.fumigatus*. Owing to the fact that both organisms share the same habitat, the production of the bacteria-specific germination inhibitor fumigermin (**104**) is considered as a fungal defense system against its bacterial competitor.

### ﻿Novel fungi and novel habitats lead to novel chemistry

**Figure 11.**
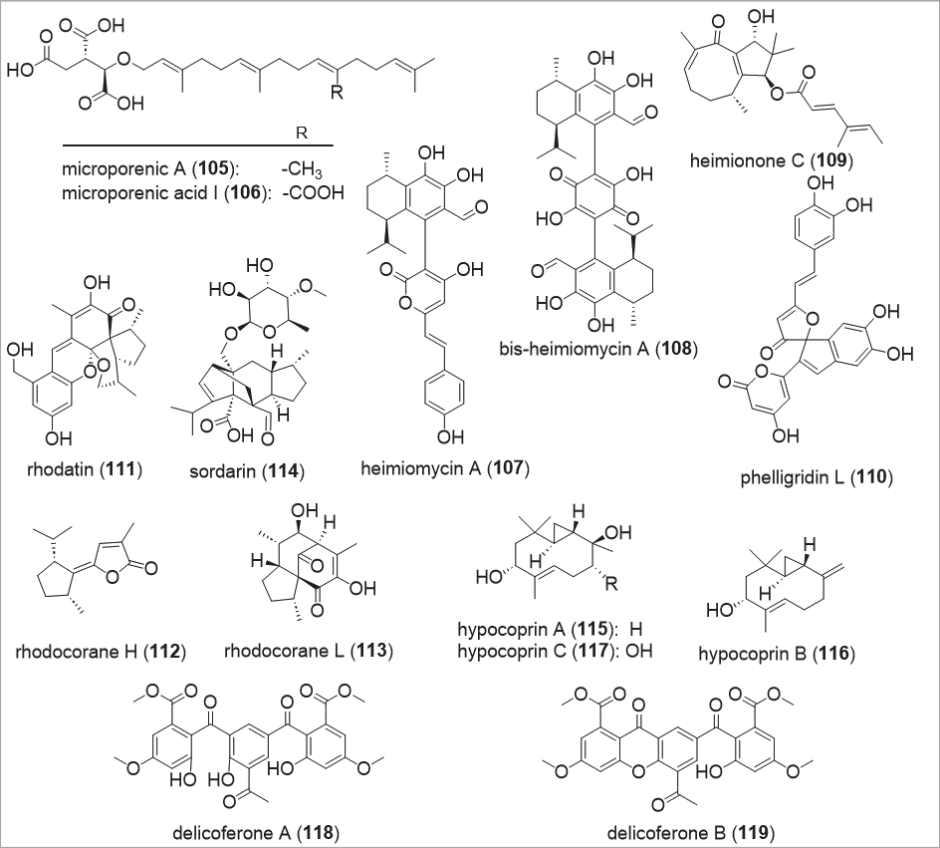
Examples for novel metabolites from under- and unexplored sources.

The discovery of novel secondary metabolites with interesting biological activities is often linked with the use of under- or unexplored species (Hyde et al. 2018). Besides untapped or difficult to handle taxa (e.g. slowly growing organisms, mycorrhizal fungi, rare taxa), sophisticated producers are frequently reported from unchartered geographical regions (e.g. the sub- or tropical regions) or unexplored habitats (e.g. fungi isolated from animal dung, particularly from herbivorous mammals). Investigations on organisms from the tropical Kenyan rain forest resulted in the discovery of many new species together with a variety of novel structurally diverse secondary metabolites. Microporenic acids (**105**–**106**), isocitric acid derivatives with polyisoprene moieties from genera of the *Polyporaceae*, namely *Microporus* sp. and Lentinuscf.sajor-caju, have been isolated as promising inhibitors of *Staphylococcusaureus* biofilms with effects within a non-lethal range for the opportunistic pathogen (Chepkirui et al. 2018a; Zeng et al. 2024; Fig. 11). When treated in combination with vancomycin and gentamycin, microporenic acid I (**106**) was able to enhance the efficacy of the established antibiotics in biofilms, indicating potential applications in combinatorial therapy. On the other hand, the isolation of several novel core structures from a new tropical *Heimiomyces* sp. is an outstanding example of the structural diversity and complexity that can prevail in a single strain. Recently, heimiocalamenes, heimiomycins (**107**), bis-heimiomycenes (**108**) or heimionones (**109**) – with a new meroterpenoid scaffold – were discovered via a study of this strain, which produced entirely different metabolite profiles in different culture media (Pfütze et al. 2023a, 2023b), with fermentation times of up to 7 months in solid state medium. Another example depicts the nematicidal phelligridin L (**110**), reported from a hitherto undescribed African species of the genus *Sanghuangporus* belonging to the *Inonotusluteus* complex, a complex otherwise well-known from Asian countries (Chepkirui et al. 2018b). Its Asian members have elaborately been studied for their chemical constituents and pharmaceutical properties due to their usage as medicinal mushrooms (De Silva et al. 2013; Cheng et al. 2019). The discovery of phelligridin L (**110**) from an African *Sanghuangporus* sp. underpins the potential of discovering novel secondary metabolites from undescribed species or unexplored regions. Another compelling and rewarding example of innovative chemistry derives from the rare temperate mushroom *Rhodotuspalmatus*. Here, the unique meroterpenoid rhodatin (**111**) and its strong antiviral activity against hepatitis C virus together with several other new sesquiterpenoid scaffolds (**112**–**113**) were discovered during a first study on its secondary metabolism (Fig. 13, Sandargo et al. 2019b, 2019c). Remarkably, rhodocorane scaffolds **112** and **113**, amongst others, were previously only known as intermediates from synthetic routes and not described as occurring in nature. Further recent examples for new chemistry from *Basidiomycota* are summarized in the review by Sum et al. (2023) and therefore will not be discussed in detail here.

Coprophilous fungi represent another promising source for chemical innovation and novel secondary metabolites. Coprophilous fungi are dung-colonizing organisms and may belong to the orders *Eurotiales*, *Hypocreales*, *Onygenales*, *Pezizales*, *Pleosporales*, *Microascales*, *Sordariales*, or *Xylariales* (Bills et al. 2013). Because of spending their complete life cycle in the dung, they are highly adapted towards their environment. Within these microcosms, coprophilous fungi are constantly challenged by a highly competitive community: Due to niche overlap with other bacteria, protists, invertebrates, the mammalian digestive system, and other fungi, they have to compete in a nutrient-rich substrate ensuring their survival and reproduction. Even if competing successfully, sought-after assimilated nutrients are now concentrating in fungal hyphae, evoking the attack of predators and parasites. Stimulated by the surrounded biodiversity, coprophilous fungi are prolific producers of numerous antimicrobial compounds and robust secondary metabolite arsenals as reviewed by Bills et al. (2013). Examples are the production of the tetracyclic diterpenoid sordarin (**114**) and derivatives thereof with strong antifungal activities from *Podosporapleiospora* isolated from rabbit pellets (Weber et al. 2005), the discovery of the sesquiterpenoids hypocoprins A-C (**115**–**117**) from *Hyprocoprarostrata* from horse dung with moderate antibacterial effects against Gram-positive germs (Jayanetti et al. 2015), or the benzophenones delicoferones (**118**–**119**) from *Delitschiaconfertaspora* from rock hyrax dung (Jayanetti et al. 2017). Studies on the coprophilous community have demonstrated that the dung habitat is characterized by a rich density of microfungi with highly significant differences regarding their seasonal occurrence, latitudinal gradient, and preferred substrate composition (Richardson 2001). Coprophilous fungi also live in strong competition with other fungi, as well as with bacteria and invertebrate animals. Compared to the high biodiversity that can be found in this habitat, along with the relatively high hit rate for novel compounds in the few studies that have so far been conducted, dung-inhabiting fungi clearly constitute an underexplored source for the discovery of new bioactive secondary metabolites (Bills et al. 2013; Charria-Girón et al. 2022).

A well-developed secondary metabolism is, however, not spread throughout all fungal groups and seems to be reserved only to specific evolutionary lineages, with the *Ascomycota* and *Basidiomycota* featuring the most prolific sources (Bills and Gloer 2016). In the next section, we want to highlight a brief selection of well-studied groups and species of these two phyla.

### ﻿Notable examples from *Ascomycota* and *Basidiomycota*

**Figure 12.**
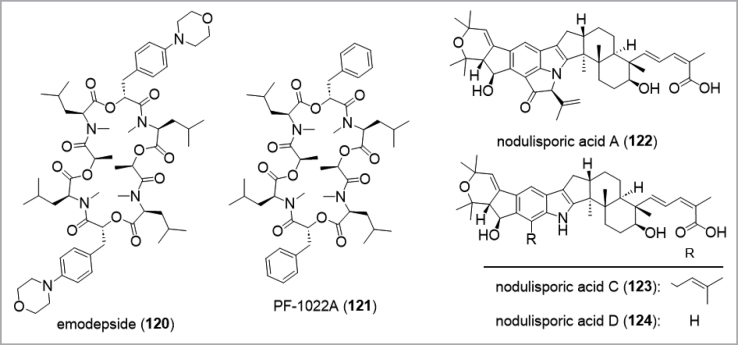
Examples for secondary metabolites isolated from *Ascomycota*.

**Figure 13.**
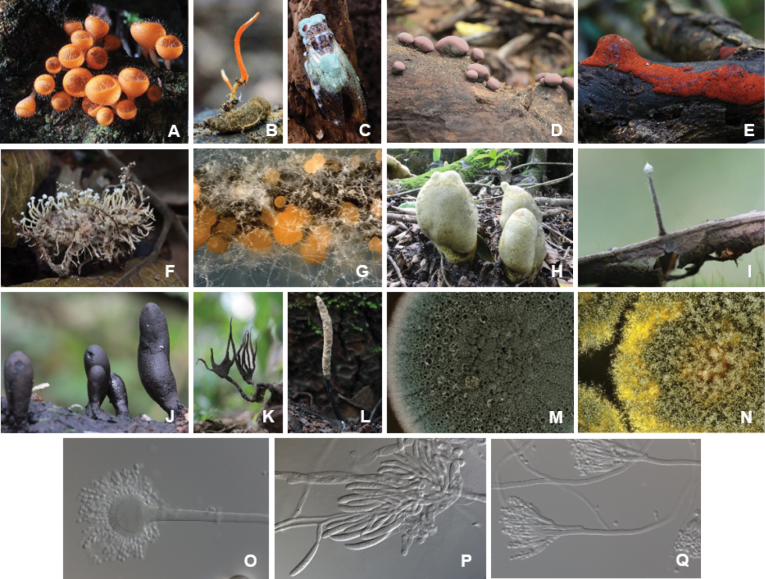
Morphological diversity of *Ascomycota*. *Cookeinatricholoma* (**A**), *Blackwellomyces* sp. on *Lepidopterapupa* (**B**), *Metarhizium* sp. on adult cicada (**C**), *Daldiniaeschscholtzii* (**D**), *Hypoxylonhaematostroma* (**E**), *Beauverialeioensis* (**F**), *Fusarium* sp. (**G**), *Squamotuberaleratii* (**H**), *Xylaria* sp. (**I**), *Xylariacubensis* (**J**), *Xylaria* spp. (**K, L**), *Penicilliumexpansum* in culture (**M**), *Aspergilluschevalieri* in culture (**N**), anamorph structures of *Aspergilluschevalieri* (**O**), anamorph structures of *Fusariumredolens* (**P**), anamorph structures of *Penicilliumexpansum* (**Q**). Photos: courtesy of NBT Plant Microbe Bank, National Biobank of Thailand, National Center for Genetic Engineering and Biotechnology, Thailand (**A, D, E, H–L**); courtesy of Plant Microbe Interaction Research Team, National Center for Genetic Engineering and Biotechnology, Thailand (**B, C, F**); Cobus Visagie (**G, M–Q**).

The *Ascomycota* are arguably the most intensely studied phylum in respect to their biodiversity among the kingdom of fungi (Fig. 13). Natural products isolated from these fungi have been extensively reviewed. Hence, the reader is directed towards reviews covering the most species-rich classes *Eurotiales* within the *Eurotiomycetes* (see also taxonomical tool section; Lan and Wu 2020), the *Hypocreales* (Wei and Wu 2020; Zhang et al. 2020; Kuephadungphan et al. 2021), *Xylariales* (Helaly et al. 2018; Becker and Stadler 2021; Kuephadungphan et al. 2021), *Amphisphaeriales* (e.g. Wang et al. 2012; Ortega et al. 2021), *Diaporthales* (Chepkirui and Stadler 2017), *Sordariales* (Charria-Girón et al. 2022) from the *Sordariomycetes* and *Lecanoromycetes* (Jahn et al. 2017; Keller 2019) as well as the *Dothideomycetes* (Stergiopoulos et al. 2013; Muria-Gonzalez et al. 2015). An example of a drug lead developed from this group is the nematicide emodepside (**118**) (Willson et al. 2003) which is a semisynthetic derivative of PF1022A (**119**), a cyclooctadepsipeptides produced by *Rosellinia* spp. (Wittstein et al. 2020; Fig. 12). Other compounds such as nodulisporic acids (**120**–**122**) and sordarin (**112**) are leads in development for their antiparasitic and antifungal properties, respectively.

Other well-known and extensively used compounds in science comprise the cytochalasans (**12**–**14**) produced by various genera of ascomycetes, for which well over hundred different structures are known. The best studied examples in regard to their bioactivity are cytochalasins B and D (**13**–**14**), which will be summarized later. A recently published review also highlighted the importance of international collaborative efforts to cartograph the enormous wealth of extractable secondary metabolites, exemplified by Thai ascomycete mycodiversity (Kuephadungphan et al. 2021).

The *Basidiomycota* include most of the mushroom-forming fungi (Fig. 15) and are the second largest division in the kingdom Fungi next to the *Ascomycota* (Wijayawardene et al. 2020). The structural variety of secondary metabolites derived from *Basidiomycota* (Sandargo et al. 2019a) and their complex repertoire of natural product biosynthesis has recently been reviewed (Gressler et al. 2021). The secondary metabolism of their mycelia and corresponding fruitbodies is complementary, and many *Basidiomycota* are prolific producers of secondary metabolites. In natural habitats, both parts have different ecological functions (Spiteller 2008). While the mycelia compete with other organisms for nutrition and space, the fruiting bodies are mostly short-living phenomena that ensure the reproduction of the producing fungus. However, the few studies available demonstrate that the corresponding mycelial cultures do at least not overproduce the constituents of the fruiting bodies. For instance, in the case of the saprotrophic genus *Hericium*, the meroterpenoids of the hericenone type (e.g. Wittstein et al. 2016) are prevailing in the fruiting bodies, while the cultures predominantly produce cyathane type diterpenoids (Rupcic et al. 2018). A recent study embarking on two of the few species of the *Boletaceae* has shown that it is possible to produce the colorful pigments (Fig. 3), such as xerocomic acid (**43**), variegatic acid (**44**), or variegatorubin (**45**), that are generally prevailing in the fruiting bodies of these fungi also in mycelial culture (Chuankid et al. 2020).

**Figure 14.**
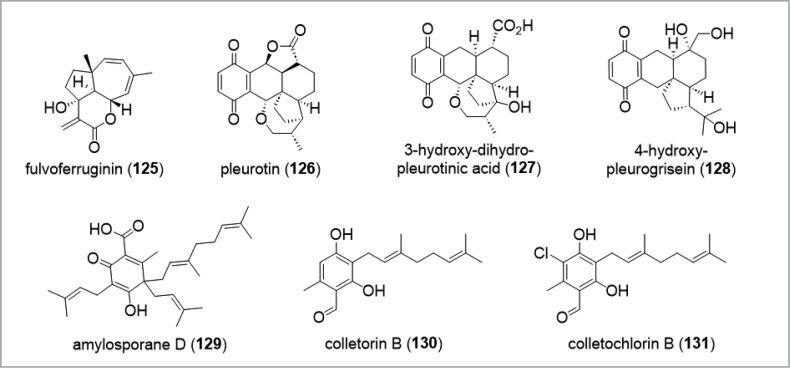
Examples for secondary metabolites from *Basidiomycota*.

In contrast to the “low hanging fruits” from soil-inhibiting molds and bacteria that have been harvested to the benefit of mankind, studying the secondary metabolism of *Basidiomycota* can be rather demanding. On the one hand, certain promising metabolites such as the anti-biofilm metabolite microporenic acid A (**105**) (Chepkirui et al. 2018), the potential cytotoxic agent fulvoferruginin (**125**) (Sandargo et al. 2021), or the antibiotic and antiviral pleurotin type meroterpenoids (**126**–**128**) from the nematophagus basidiomycete *Hohenbueheliagrisea* (Sandargo et al. 2018; Fig. 14) were fairly well accessible with yields of several hundred mg per liter without any extensive need to optimize the production of the wild type strains. On the other hand, there are many other species of *Basidiomycota* that take up to several months to grow under regular culture conditions, and there are many others that cannot be cultured at present. This is probably due to the fact that these species rely on symbiotic relationships in their natural habitats or have other, hitherto unknown nutrient requirements. The polypore of the genus *Amylosporus* are associated with grasses (Campi et al. 2017), and took almost 3 months of growth in liquid culture for the production of amylosporanes (**129**) and the antibacterial agents colletorin B (**130**) and colletochlorin B (**131**) (Matio Kemkuignou et al. 2022). Conceivably, systematic biotechnological exploitation of *Basidiomycota* can hence be even more difficult than for other *Ascomycota* due to their slow growth or low production titers.

**Figure 15.**
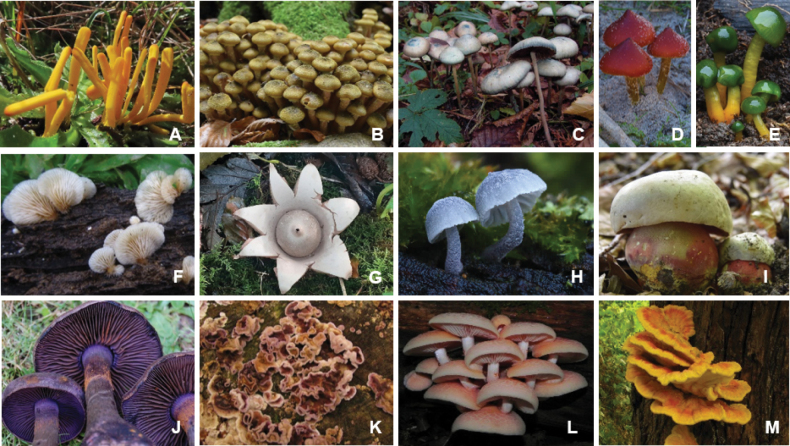
Morphological diversity of *Basidiomycota*: *Clavulinopsis* sp. (**A**), *Armillariamellea* (**B**), *Psilocybecyanescens* (**C**), *Hygrocybeconica* (**D**), *Gliophoruspsittacinus* (**E**), *Hohenbuehelia* sp. (**F**), *Geastrummichelianum* (**G**), *Mycenapseudocorticula* (**H**), *Rubroboletussatanas* (**I**), *Cortinariusviolaceus* (**J**), *Chondrostereumpurpureum* (**K**), *Rhodotuspalmatus* (**L**), *Laetiporussulphureus* (**M**). Photos: Harry Andersson (**C, K, M**); Benjarong Karbowy-Thongbai (**F**); Torsten Richter (**B, D, E, H–J**), Hans Pfeiffer (**A**); Hedda Schrey (**G**); Jürgen Schnieber (**L**).

### ﻿Secondary metabolites as taxonomical tools in the systematics of fungi

Secondary metabolites can occur in the form of conspicuous pigments, where they can exhibit useful properties for chemotaxonomical approaches (summarized by Frisvad et al. 2008). This system has successfully been used to reorder the systematics of species, genus, or even families in the Kingdom Fungi, both in the division *Basidiomycota*, particular in the *Boletales* (Gill and Lally 1985; Winner et al. 2004; Bresinsky 2014) and in the *Ascomycota* (e.g. in *Aspergillus*, *Penicillium* and the *Hypoxylaceae*). The key concept lays in the combination of different phenotypic characters, such as morphology, chemical constituents and multilocus genetic data in polyphasic approaches.

Interest to achieve metabolic profiling of *Aspergillus* spp. and *Penicillium* spp. (a genus which was eventually segregated into the genera *Penicilliums. str.* and *Talaromyces*, also based on chemotaxonomic criteria) is strongly linked to their importance as mycotoxin producers as both food related molds and human pathogens and due to their widespread usage as biotechnological workhorses for the production of enzymes, citric acid and in food industry. Domesticated *Aspergillus* species feature, for example, *A.niger*, A.flavusvar.oryzae and *A.sojae*. The taxon *A.niger* is classified in section Niger, while A.flavusvar.oryzae and *A.sojae* are classified in section Flavi, two sections known to feature potent mycotoxin producers. Hence, metabolic profiling and a thorough taxonomic characterization may contribute towards minimizing the risk of using mycotoxigenic fungal strains in industrial application (reviewed by Houbraken et al. 2014 and Frisvad et al. 2018). In the clinical context, it is understandably of high relevance to reliably tell if an *Aspergillus* infection coincides with production of the potent aflatoxins (**132**–**135**) or immune suppressive gliotoxins (**96**). Knowledge of these traits has serious implications for the prospect of treatment options for patients. Metabolic profiling of *Aspergillus*, but also *Penicillium* spp. by HPLC coupled to an UV-Vis detection system was shown to be feasible for chemotyping of isolated cultures in 1989 by Frisvad and turned out to be a highly consistent phenotypical character for taxonomic purposes (taxonomic overview by Houbraken et al. 2020). The enormous wealth of secondary metabolites (termed extrolites in *Aspergillus* and *Penicillium* taxonomy, as being ‘outward’ directed chemicals) described for the different systematic sections is in the process of being reviewed extensively (Frisvad and Samson 2004; Samson et al. 2004; Frisvad et al. 2007; Nielsen et al. 2009; Frisvad and Larsen 2015, 2016; Kocsubé et al. 2016; Frisvad et al. 2019; Ráduly et al. 2020) in toxicological and taxonomic contexts. Among the 807 secondary metabolites described until 2017 (Vadlapudi et al. 2017), many substance classes emerged as being taxonomically informative to improve or support species descriptions in combination with other observations in polyphasic approaches. This was last assessed comprehensively by Kocsubé et al. (2016) to settle the monophyly of *Aspergillus* segregated from *Penicillium*.

**Figure 16.**
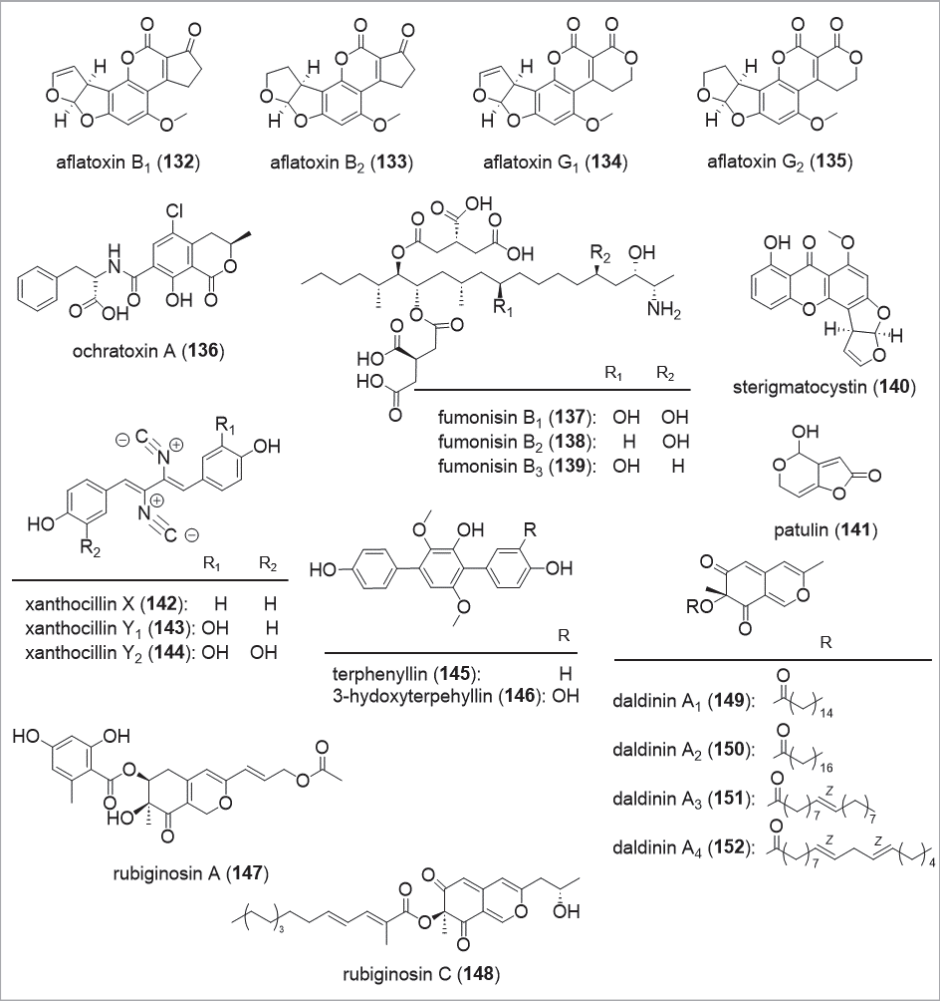
Examples for chemotaxonomic markers for *Ascomycota*.

Strong public interest is focused on their relevance as mycotoxin producers, which account for huge economic losses by food spoilage, but also for public health due to contaminated food (Ráduly et al. 2020). The most important toxins from *Aspergillaceae* are the aflatoxins (especially of type B_1_, B_2_, G_1_ and G_2_, **132**–**135**). Ochratoxin A (**136**) and gliotoxin (**96**) are also common in *Aspergillus*, while fumonisins (**137**–**139**) occur occasionally in *Aspergillus* but mostly in *Fusarium* spp. and sterigmatocystin (**140**) is widespread in *Aspergillus* but even occurs in many other genera like *Chaetomium* (Rank et al. 2011). Aflatoxins (especially produced by AspergillussectionFlavi) are carcinogenic and can lead to death in acute intoxication events (Dhanasekaran et al. 2011). Ochratoxin A (**136**) (present in both *Aspergillus* and *Penicillium*) has a wide range of toxic effects on the human body, while carcinogenic properties are being hypothesized as it can induce cancer in animal model systems (Heussner et al. 2015). Gliotoxin (**96**), typically produced by Aspergillussect.Fumigati, is often referred to as a virulence factor, playing an important role in clinical infections, suppressing the host’s immune response. However, not every producing strain has also been shown to possess human pathogenic tendencies (Corrier et al. 1991; Frisvad and Larsen 2016). Sterigmatocystin is biosynthetically very similar to aflatoxins and can even be converted when an aflatoxin producing competent and deficient *Aspergillus* co-colonize the same substrate (EFSA 2013). While still being toxic, its carcinogenic potential is far lower than that of aflatoxins (**132**–**135**; Fig. 16). Fumonisins (**137**–**139**) are also well known to exert carcinogenic potencies and to induce developmental disorders like defects in the neural tube and toxicity against kidney and liver (Nair 1998). Patulin (**141**) is another well-known mycotoxin, which typically occurs in *Penicilliumexpansum* but occasionally also occur in other Penicilia and even Aspergilli (subgenera *Aspergillus*, *Cremei* and *Fumigati*). It can frequently be found in apple juice derived from moldy apples (Frisvad 2018). Yeasts, however, are able to break down the compound during fermentation (Yu et al. 2007), making the ingestion of cider comparably safe (disregarding the chance of alcohol poisoning). Other more broadly distributed secondary metabolites are the xanthocillins (**142**–**144**) and terphenyllins (**145**–**146**), which are evenly distributed among all subgenera of *Aspergillus*.

A chemotaxonomic classification also helped to resolve many taxonomical issues in the important xylarialean family *Hypoxylaceae*. Here, a polyphasic approach combining chemical, genetical and a morphological analysis of environmental samples from saprobically growing teleomorphic structures of genera like *Annulohypoxylon* (Kuhnert et al. 2017), *Daldinia* (Stadler et al. 2014) and *Hypoxylon* (Kuhnert et al. 2014; Sir et al. 2016) helped to settle many incongruent classifications based on morphological data alone. Members of the *Hypoxylaceae* often contain large amounts of secondary metabolites in their wood-inhabiting stromata, which may even exceed 10% of the total dry biomass (Stadler and Fournier 2006; Stadler et al. 2007). These compounds are mostly azaphilones (e.g. rubiginosins (**147**–**148**), mitorubrins (**54**) and daldinins (**149**–**152**), but also compounds primarily associated with younger growth stages (e.g. cytochalasins **12**–**14).** While these compounds proved to be of value as chemical markers, their precise role in nature is comparably poorly understood. In the case of rubiginosin C (**148**) it was found that the fungal pigment is able to interfere with the formation of biofilms and the yeast-to-hyphae transition of *Candidaalbicans* and *Candidozymaauris*. This morphological change is an important driver for the establishment of stable and resistant biofilms on surfaces (Zeng et al. 2023), a potential indicator for its ecological function. Nevertheless, most of the compounds found in stromal extracts cannot be produced by fermentation. Hence, availability is currently restricting biotechnological exploitation (Becker and Stadler 2021). However, as the genomic era is more and more introduced into fungal secondary metabolite research, comparative genomic studies may enable exploiting chemotaxonomical information by linking compound production to the presence of specific BGCs detectable in different phylogenetic clusters and improve systematics by using phylogenomics, in turn again fostering the identification and prediction of biosynthetic gene clusters in sequenced genomes (Kuhnert et al. 2021; Wibberg et al. 2021).

Other approaches include the analysis of the protein composition *via*MALDI-TOF, used as a rapid identification method in a clinical context, which is very helpful for diagnostics of human pathogens and far superior over the ITS barcoding approaches that often have little discriminatory power (Bader 2017; Becker et al. 2019). Its versatility and complementarity have recently been demonstrated for the zoonotic fungal pathogen species complex *Trichophyton*, where its diagnostic feasibility was validated by a detailed phenotypical study including morphology, genetical information and microsatellite marker analysis (Čmoková et al. 2020). Notably, MALDI-TOF is a proteomics-based technique that has nothing to do with secondary metabolite analysis. In a recent study on *Pyrenopolyporus* from Thailand, MALDI-TOF was also found suitable to resolve a complicated species group (Wongkanoun et al. 2023). However, the effort to create the analytical data after standardized cultivation and analytics hitherto was found to be much more strenuous than the more conventional approach using morphology, molecular phylogeny and HPLC profiling, and it requires availability of viable cultures. Therefore we strongly advise against its broad use outside the medical field.

### ﻿Secondary metabolites as biochemical tools

While only few fungal metabolites have made it to the pharmaceutical market or inspired the development of synthetic drugs, the number of biosynthetic tool compounds that are valuable in biochemistry, cell biology, physiology and related disciplines is much higher. Not every natural product has optimal chemical and physical characteristics to serve as a potential new drug. However, once the mode of action is characterized, secondary metabolites can become attractive tools to track or interfere with specific biological processes.

**Figure 17.**
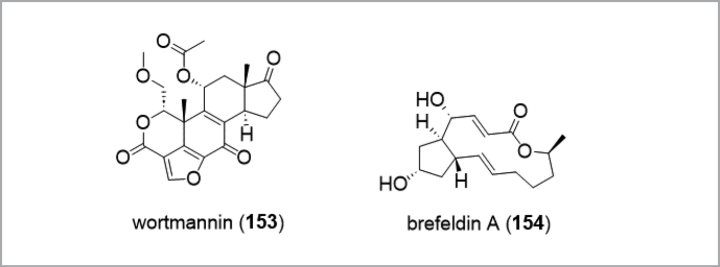
Examples of secondary metabolites from fungi which are used as biological tools in science.

Wortmannin (**153**; Fig. 17), for example, is a furanosteroid first isolated from *Penicilliumwortmannii* (now *Talaromyceswortmannii*) as antifungal agent (Brian et al. 1957). Organismic cytotoxic hemorrhagic effects on rats were noticed by Abbas and Mirocha (1988). In studies involving neutrophils, wortmannin (**153**) and related compounds were shown to inhibit the respiratory burst, an immunological response to phagocytosis generating vast amounts of reactive oxygen species to kill-off taken up particles (Baggiolini et al. 1987). Exploration of the signal cascade responsible for the neutrophil response indicated the involvement of two G-protein mediated cascades (Dewald et al. 1988), which led to the description of wortmannin (**153**) as a phosphatidylinositol 3-kinase (PI3K) inhibitor in the low nanomolar range (Arcaro and Wymann 1993). Its selectivity for the PI3K enzyme was later assessed by Powis et al. (1994) and its mode of action elucidated by Wymann et al. (1996), however, Liu et al. (2005) could show that the mammalian polo-like kinase (PLK) poses an additional cellular target, undermining the previous thought of wortmannin representing a selective inhibitor of the PI3K. This finding gave the compound implications as an anti-cancer agent, as PLK has been shown to be overexpressed in various cancers (Strebhardt 2010), in addition to blocking the signal transduction to enable DNA repair in response to DNA damage in yeast (Zewail et al. 2003). However, compound stability issues limited its potential use as reviewed by Wipf and Halter (2005). Advances in drug delivery systems assessed and discussed by Karve et al. (2012) may clear the way for its potential use as a radiosensitizer if its systemic toxicity can be handled, however, only time will tell if this new direction can spark new interest in exploring its capacities in the medicinal context. Nevertheless, this knowledge was of great help to access the role of PI3K not only in mammals, but also in yeast and plants, where it was used to better understand and study vesicle trafficking (Zewail et al. 2003; Wang et al. 2009; Takáč et al. 2012; Liu et al. 2020b).

Another broadly applied secondary metabolite is brefeldin A (**154**) formerly described as decumbin, cyanein, ascotoxin, synergisidin or nectrolide), a macrocyclic lactone exhibiting antiviral, cytotoxic, phytotoxic and cancerostatic effects, as well as effects on fungal morphogenesis (reviewed by Betina 1992). First isolated as decumbin (Singleton et al. 1958) from *Penicilliumdecumbens* and later formally described as brefeldin A (**154**) from *P.brefeldianum* (Härri et al. 1963; Sigg 1964; Singleton and Bohonos 1964), it is best known for its inhibitory effect on the protein sorting machinery associated with the golgi apparatus in animal and plant cells (reviewed by Nebenführ et al. 2002). Brefeldin A (**154**) became of particular importance due to its ability to block intracellular transport (Misumi et al. 1986) and cause disassembly of the Golgi apparatus and its fusion with the endoplasmatic reticulum. This ultimately led to the description of the retrograde membrane trafficking pathway from the cis- side of the Golgi back to the endoplasmatic reticulum (Lippincott-Schwartz et al. 1989; Klausner et al. 1992). Brefeldin A (**154**) thus played a major role in deciphering membrane traffic and secretion pathways (reviewed by Pelham 1991; Klausner et al. 1992; Chardin and McCormick 1999), far before its intracellular target has been identified (Arf guanine nucleotide exchange factors, GEFs; see Niu et al. 2005). Since then, it is now well defined as inhibitor of coating-protein assembly enabling the formation of vesicles and most commonly discussed in the context of Arf-GEF interaction (reviewed by Jackson 2018; Walton et al. 2020).

The last example comprises phalloidin (**81**) from *Amanitaphalloides* and cytochalasins (e.g., **12**–**14**), which frequently occur in the orders *Eurotiales*, *Sordariales* and *Xylariales* (Scherlach et al. 2010; Becker and Stadler 2021; Charria-Girón et al. 2022), amongst others. These inhibitors are well known to interfere with the eukaryotic actin cytoskeleton but differ in their mode of action. Phalloidin (**81**) acts as a stabilizer of filamentous actin structures, while cytochalasins have been described to inhibit F-actin polymerization among other actin and non-actin related effects (Copper 1987; Sampath and Pollard 1991). Phalloidin (**81**) has mostly been used in its early days to study the role of a disrupted actin cytoskeleton due to excessive stabilization, which made it a very valuable tool to study actin structures back when the role of actin itself was not conclusively established (cf. Wehland et al. 1977). Later, its tight and rather selective association with polymerized actin was exploited to develop an easy-to use fluorescent probe to visualize F-actin structures, which gave rise to an alternative actin staining tool besides the use of actin antibodies for cell biologists (Wulf et al. 1979; reviewed by Faulstich et al. 1988), even before phalloidins’ precise biochemistry and mechanism of action was comprehensively understood (Coluccio and Tilney 1984; Vandekerckhove et al. 1985; Barden et al. 1987; Sampath and Pollard 1991). Even though it was known for a long time, recent developments still increased our understanding of the chemistry of phalloidin (**81**) (Yao et al. 2019). Nevertheless, its role in microscopical high-end super resolution imaging will at some point likely be replaced by other techniques that are currently in development (cf. Mazloom-Farsibaf et al. 2021). Cytochalasins are best known for their interference with actin polymerization by inhibiting monomer addition (**12**–**14**), but also other cellular targets have been described (see Kapoor et al. 2016). They are specifically used in literature to study the role of active (or inactivated) actin polymerization in cellular movement or actin associated processes. From the hundreds of hitherto described cytochalasan related structures (Zhu et al. 2021), cytochalasins B and D (**13**–**14**) can be highlighted as the most frequently encountered molecules (cf. Cooper 1987; Van Goietsenoven et al. 2011; Lambert et al. 2023). In the early days of actin and motility research, cytochalasins (**12**–**14**) played a major role in attributing filament growth in the neuronal growth cone to actin. Usage of cytochalasins to investigate and inhibit contractile ring formation during cell division led to an analogous conclusion, however, a surprising one at that time, that nuclear division was not inhibited. This simultaneously demonstrated the independence of nuclear and cell division from one another, summarized by Peterson et al. (2002) as hallmark achievements using these compounds. Actin as cytochalasan’s prominent cellular target was only comprehensively described later (Schroeder 1970; Spudich and Lin 1972; Ohmori et al. 1992). Apart from detailed studies on selected compounds, the impact of chemical differences in the core cytochalasan structure is not comprehensively understood (Scherlach et al. 2010), despite several studies attempting to gain knowledge by screening several cytochalasins (Yahara et al. 1982; Van Goietsenoven et al. 2011; Kretz et al. 2017). There is much more to learn about potential fields of uses, as recent papers show much potential in modifying and outlining differential effects for other cell biological (or drug-related) applications (Skellam 2017; Wang et al. 2019a; Moussa et al. 2020; Wang et al. 2020; Lambert et al. 2021). For further reading, we would like to direct the inclined reader to our recent review on the paper, where we have summarized cytochalasans’ impact on actin filament remodeling in more detail (Lambert et al. 2023).

## ﻿Synthetic biology approaches to natural product chemistry

Recent studies in the Genomics era have revealed that fungal genomes contain an unexpected number of BGC that does not match the number of secondary metabolites previously reported from these organisms. This phenomenon is usually referred to as the presence of “silent” metabolic pathways that need to be activated (Keller 2019). We here just mention some prominent examples of how researchers have tried to tackle this challenge. This can be achieved by molecular genetic manipulations, such as gene deletion (knockouts) or by expression of a set of BGC-associated genes in a different organism (heterologous gene expression, Krappmann 2014; Lazarus et al. 2014; He et al. 2018). This concurrently allows the functional dissection of associated genes and enzymes involved. Challenges involve the selection of suitable host strains that allow the correct expression of target genes and that produced compounds are non-toxic for the host (Markina et al. 2020) – which can sometimes be solved by including additional genes of the associated BGC, as gene clusters were frequently shown to carry self-resistance genes (Keller 2015; Zhang et al. 2021). Moreover, potential chemical modification of host – or even the native producers themselves – concerning enzymatic crosstalk modifying the final product or shunts of intermediates produced during biosynthesis need to be considered (Kjærbølling et al. 2019).

In order to identify and predict BGCs from genomic data, the most frequently used data analysis pipeline is composed of the anti-SMASH (Blin et al. 2023). This analysis suite is continually developed to improve BGC detection, transcription factor and even chemical structure prediction. Notably, gene cluster prediction depends on the quality of the genome sequence data, for which sequencing technology platforms such as offered by Oxford Nanopore or Pacific Biosciences seem to be more than suited for (Kuhnert et al. 2021; Wibberg et al. 2021). With increasing data amount, this also opens avenues to study secondary metabolite gene cluster evolution in larger population sets in unprecedented detail (discussed above for the *Hypoxylaceae*; Kuhnert and Collemare 2022). Moreover, this will further deepen our knowledge concerning the chemical and enzymatical logic of fungal assembly lines (for several excellent reviews and book, see Cox 2007; Cox 2014; Matsuda et al. 2016; Walsh and Tang 2017; Schor and Cox 2018; Kahlert et al. 2021), at some point maybe enabling the design of completely new natural products.

## ﻿Exploiting the biosynthetic machinery to increase chemical space: Mutasynthesis and rational design

Synthetic chemists often struggle to recreate the complex chemistry employed by nature from scratch utilizing basic building blocks, but may sometimes be able to utilize similar strategies in a biomimetic fashion (such as for the synthesis of Sch-642305, see Snider and Zhou 2006). Fungal chemistry is also discussed as a tool to further increase chemical diversity by employing their biosynthetic (‘mycosynthetic’) potential in tandem with traditional total- and semi-synthesis (Kahlert et al. 2021). Indeed, further work on honing these strategies will give rise to new possibilities to create new chemistry and facilitate systematical re-creation and diversification of compound synthesis in a rational fashion in heterologous hosts. For a more comprehensive overview on the latter topic, we refer to the recent review by Cox (2024). The next paragraph highlights a few examples for potential application of such strategies.

**Figure 18.**
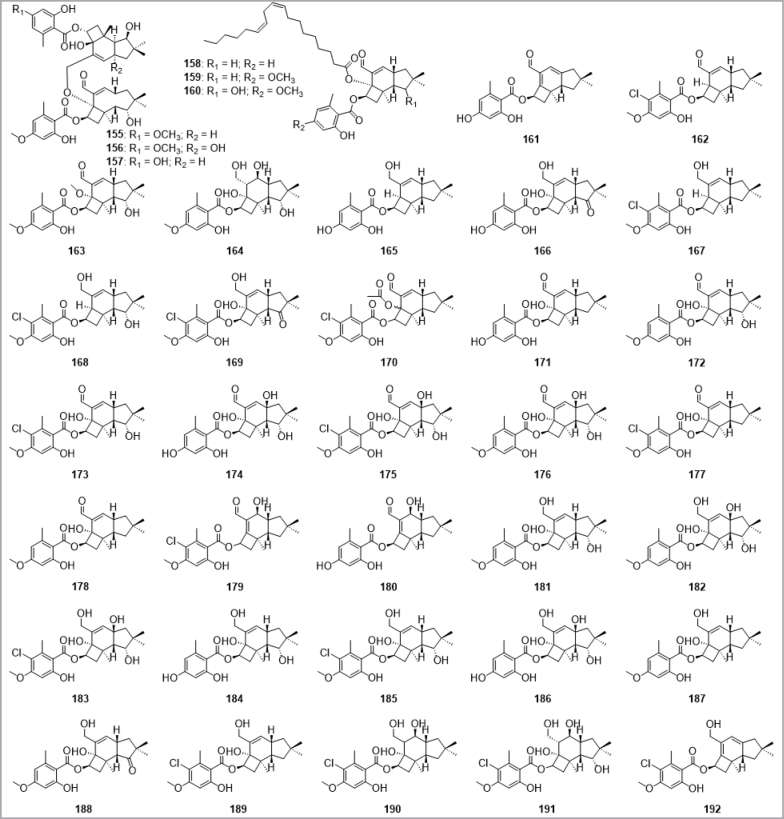
Melleolide-type meroterpenoids described by Pfütze et al. (2024). Compound numbers correspond to bismelleolide BH-CH and EH (**155–157**); melleolide linoleate (**158**); armillarine linoleate (**159**); melleolide H linoleate (**160**); 5’-O-desmethylarmillaribin (**161**); 4-dehydroxyarmillaridin (**162**); 4-methoxymelleolide H (**163**); 10-hydroxy-5’-O-methylarmillane (**164**); 4-dehydroxymelleolide F (**165**); 10-ketomelleolide E (**166**); 4,10-dehydroxymelleolide I (**167**); 4-dehydroxymelleolide I (**168**); 10-ketomelleolide I (**169**); 4-acetylarmillaridin (**170**); melleolide (**171**); melleolides H and J (**172**–**173**); melledonals A and C (**174**–**175**); 5’-O-methylmelledonal (**176**); armillaridin (**177**), armillarin (**178**); arnamial (**179**); dehydroarmillylorselinate (**180**); melleolides B-E and I (**181**–**185**); melledonol (**186**); 10-dehydroxy-melleolide B (**187**); 10-oxo-melleolide B (**188**); A52a (**189**); 5’-methoxy-6’-choloroarmillane (**190**); 10-hydroxy-5’-methoxy-6’-chloroarmillane (**191**); 1-hydroarmillaricin (**192**). The biosynthesis of melleolides was recently addressed by Fukaya et al. (2023).

As discussed further above, biosynthetic routes have frequently been observed to exhibit cross-talk or intersect with other biosynthetic routes, combining building blocks of differing origin in one compound. Well-studied examples can be found among meroterpenoids or NRP-PKs, which show an astonishing degree of versatility (e.g. Wasil et al. 2013; Matsuda and Abe 2016; Skellam 2017). Meroterpenoids, as terpenoids themselves, are comprised of isoprene units of different lengths, which are added to a given backbone (Walsh and Tang 2017). Meroterpenoids are arguably among the most complex natural products synthesized by fungi (Nazir et al. 2021). Recent advances on the biochemistry of meroterpenoid cyclases highlighted a surprising promiscuity of the enzymes involved (Mitsuhashi et al. 2020): Selected cyclases were tested for their substrate scope on a set of natural and unnatural meroterpenoids, which were complemented with further studies on their reaction kinetics. This led to the discovery of 12 new complex unnatural chemical scaffolds. Another example for bioactive meroterpenoids are the melleolides from *Armillaria* species (Midland et al. 1982; Donelly et al. 1985). We recently studied the secondary metabolism of *Armillariaostoyae* by varying culture conditions and growth media and were able to isolate in total 38 different derivatives (Fig. 18, Pfütze et al. 2024). Interestingly, dimerized bismelloilides were encountered for the first time. The astonishing diversity of different congeners prompted a search for additional congeners using sophisticated cheminformatics and mass spectrometric tools. This analysis provided evidence for the presence of dozens of additional congeners in the crude extracts of a single strain. If these detectable congeners actually relate to isolatable compounds or merely to unstable intermediates, or spectroscopic artifacts remains to be shown, but a recent study paving the way towards total biosynthesis of melleloides might facilitate this process (Fukaya et al. 2023).

In comparison to meroterpenoids, NRP-PK-natural products display a remarkable degree of modularity (as gene clusters encoding PK do in general). Here, a modular PKS produces a backbone comprised of acetyl units of different degrees of saturation (low reducing or highly reducing PKS) which is coupled to amino acids generated in catabolic processes or other unusual peptides synthesized by the same, or even by cross-talk with other NRP gene clusters located in the genome. The promiscuity of a given NRPS in terms of accepting different precursors is an important parameter to enable interventions with the biosynthetic process, which opens the door for precursor-directed secondary metabolite discovery, but also manipulation by synthetic biological approaches (Wasil et al. 2013).

**Figure 19.**
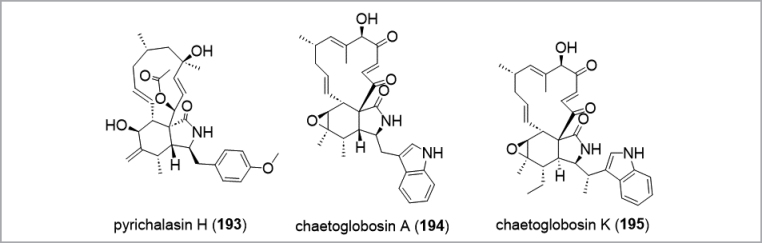
Chemical structures of pyrichalasin H (**193**), and chaetoglobosins A and K (**194**–**195**).

As mentioned previously, gene clusters encoding enzymes accepting a variety of different substrates and thus showing high degrees of promiscuity can serve as valuable exploitable targets for the rational design of compounds to increase chemical space or for specific functionalization. Pyrichalasin H (**193**; Fig. 19), as one example, is a NRPS-PKS derived natural product first described from *Pyriculariaoryzae* appearing in different plant pathogens (e.g. *Pyriculariagrisea*) of the cytochalasan family (Nukina 1987). Cytochalasans are typically comprised of an isoindole moiety, a macrocyclic ring and an amino acid. Well known cytochalasans from *Chaetomium* spp., called chaetoglobosins (**194**–**195**), typically include tryptophan as amino acid, with other common variants incorporating phenylalanine (most cytochalasins) or other amino acids containing hydrophobic side chains. A recent overview of the structural and biosynthetic variety is given by Skellam (2017).

Recently, progress has been made in the understanding of the biosynthesis of pyrichalasin H (**193**) in *Pyriculariagrisea* NI980 (Wang et al. 2019a), where a systematic knockout study on the predicted cluster led to its verification and interestingly, to the production of several new analogues of **193**. A later study confirmed that P450 oxidases involved in oxidative tailoring steps from other biosynthetic gene clusters and even other species, can reconstitute the production of the final compound in corresponding knockout strains (Wang et al. 2019b). Not all P450 oxidases could rescue pyrichalasin H (**193**) production, but instead led to the production of new cytochalasans (**12**–**14**), of which three epoxidated variants were described in the discussed study.

While these studies were more focused on the establishment of the biosynthesis and the consequences of disturbances in biosynthetic tailoring steps, Wang et al. (2020) could exploit the apparent promiscuity of the NRPS-PKS adenylation domain. Here, it could be shown by mutasynthesis that feeding halogenated phenylalanine to a knockout missing *pyiA*, an O-methyl transferase preparing phenylalanine by O-methylation for the biosynthetic incorporation is essential for the fungus to form pyrichalasin H. Wang et al. (2020) exploited these findings by feeding a 4’azido-phenylalanine precursor, assisting in subsequent semisynthetical derivatization of the compound applying click-chemistry with alkines attached to different functional groups for further mechanistic studies. This approach opened a whole toolbox of molecules for different biological and biochemical applications, for which further studies have to follow to explore their effectiveness.

## ﻿Outlook

In their natural habitats, fungi are productive and prolific producers of ingenious metabolites with potent antimicrobial activities. Modern natural product research should treasure the link between production of secondary metabolites and their ecological context, as fungi behave differently under laboratory conditions lacking external stimuli from their natural habitats. Therefore, more research on innovative strategies is needed in order to challenge fungi to reveal their full chemical arsenal. Nevertheless, determining the ecological and practical function, or biotechnological application of fungi and their natural products can be a daunting task, as a description of their biological target without preliminary knowledge is rather challenging given the predictive capabilities we have available today. Due to the complexity of the task, empirical studies, such as screening for bioactivity in different scenarios and contexts, are imperative to tackle these questions. Since the combinatorial possibilities of potential targets are endless, we strongly recommend to cooperate to cover as much ground as possible. Recently published white papers and reviews by the International Natural Product Sciences Taskforce in high ranking journals clearly show the surging interest in public on natural product research, prompting a tight connection and necessity for biologists and chemists to work together. (Atanasov et al. 2021; Miethke et al. 2021).

Reflecting the work on the description of taxa from underexplored habitats, a high degree of biodiversity has been shown to go hand in hand with chemical diversity. However, fungi which are difficult to maintain in a laboratory environment are posing a serious challenge for systematic natural product description. One strategy is composed of developing techniques focusing on reducing the ratio of previously uncultivable or slow growing fungi, e.g. mycorhizal fungi. The classical approaches to natural product discovery, i.e. systematic screening approaches, fermentation, isolation, and structure elucidation are still key assets for finding novel secondary metabolites. Alternatively, harnessing the genetic resources for the biotechnological production of secondary metabolites by heterologous gene expression out of their encoding gene clusters is a goal which can be aimed for. Scrutinizing the mechanistical rationale of genetic and enzymatic assembly machinery involved in the chemical biosynthesis enables targeted interception by mutasynthesis and opens the way for rationally designing compounds by combinatorial biosynthesis, which will be a key feat to achieve in the future to systematically explore the chemical landscape for further expenditures in biotechnological applications.

**Table 3.** Abbreviations used in this text and in natural product discovery research.

Abbreviation	Description	Abbreviation	Description
Ac-CoA	Acetyl-coenzyme A	MCD	Molecular Connectivity Diagram
Anti-SMASH	Secondary Metabolite Analysis Shell	MDLC	MultiDimensional Liquid Chromatography
BGC	Biosynthetic Gene Cluster	ML	Machine Learning
CASE	Computer Assisted Structure Elucidation	MPLC	Medium Pressur Liquid Chromatography
CCC	Countercurrent Chromatography	MRM	Multiple Reaction Monitoring
CD	Circular Dicroism	MTPA	α-methoxy-α-trifluoromethylphenylacetic acid
C/N ratio	Carbon to Nitrogen ratio	NMR	Nuclear Magnetic Resonance
COSY	Correlation Spectroscopy	NOESY	Nuclear Overhauser and Exchange Spectroscopy
CSA	Chiral Solvating Agents	NP	Normal Phase
DAD	Diode Arrac Detector	NRP	Non-Ribosomal Peptide
DESI	Desorption Electrospray Ionization	NRPS	Non-Ribosomal Peptide Synthase
ECD	Electronic Circular Dichroism	NRP-PKs	Non-Ribosomal Peptides coupled to PolyKetides
EFSA	European Food Safety Authority	1D / 2D	One Dimensional / Two Dimensional
EMA	European Medicines Agency	OSMAC	One Strain Many Compounds
ESI	Electrospray Ionization	PK	Polyketide
FCC	Flash Column Chromatography	PKS	Polyketide Synthase
GC	Gas Chromatography	QMS	Quadrupole Mass Spectrometry
GNPS	Global Natural Products Social Molecular Networking	QTOF	Quadrupole Time of Flight
HOSE	Hierarchical Organization of Spherical Environments	RDC	Residual Dipolar Coupling
HSSE	HeadSpace Sorptive Extraction	ROESY	Rotating-frame nuclear Overhauser Effect correlation spectroscopy
HPLC	High Performance Liquid Chromatography	RP	Reversed Phase
HILIC	Hydrophilic Interaction Chromatography	SCFE	Super Critical Fluid Extraction
HR-TOFMS	High-resolution Time of Flight Mass Spectrometry	SEC	Size Exclusion Chromatography
HTS	High Throughput Screening	S/N	Signal to Noise
IEC	Ion Exchange Chromatography	SPE	Solid Phase Extraction
IMS	Ion Mobility Spectrometry	SPME	Splid Phase Micro Extraction
JBCA	J-Based Configurational Analysis	TLC	Thin Layer Chromatography
LH-20	Liquid chromatography medium, properties: Lipophilic Hydrophobic, particle size 20 µm	TOCSY	Total Correlation Spectroscopy
LR-HSQMBC	Long-Range Heteronuclear Single Quantum Multiple Bond Correlation	UDB	Universal NMR database
LR-selHSQMC	Long-Range selective Heteronuclear Single Quantum Multiple Bond Correlation	UHPLC	Ultra High Performance Liquid Chromatography
MALDI	Matrix-Assisted Laser Desorption/Ionization	VOC	Volatile Organic Compounds

## ﻿Additional information

### Author ORCIDs

Christopher Lambert https://orcid.org/0000-0002-1899-8214

Marc Stadler https://orcid.org/0000-0002-7284-8671

### Data availability

All of the data that support the findings of this study are available in the main text.
